# A Novel Gene List Identifies Tumors with a Stromal-Mesenchymal Phenotype and Worse Prognosis in Gastric Cancer

**DOI:** 10.3390/cancers15113035

**Published:** 2023-06-02

**Authors:** Secil Demirkol Canli, Meral Uner, Baris Kucukkaraduman, Diren Arda Karaoglu, Aynur Isik, Nesrin Turhan, Aytekin Akyol, Ismail Gomceli, Ali Osmay Gure

**Affiliations:** 1 Molecular Pathology Application and Research Center, Hacettepe University, 06100 Ankara, Turkey; 2 Department of Molecular Biology and Genetics, Bilkent University, 06800 Ankara, Turkey; baris.blknt@gmail.com; 3 Division of Tumor Pathology, Cancer Institute, Hacettepe University, 06100 Ankara, Turkey; 4 Department of Pathology, School of Medicine, Hacettepe University, 06100 Ankara, Turkey; meral.uner@hacettepe.edu.tr (M.U.); aytekina@hacettepe.edu.tr (A.A.); 5 Faculty of Medicine, Hacettepe University, 06100 Ankara, Turkey; ardakaraoglu96@gmail.com; 6 Hacettepe University Transgenic Animal Technologies Research and Application Center, 06100 Ankara, Turkey; aynur.isik@hacettepe.edu.tr; 7 Ankara City Hospital, Department of Pathology, University of Health Sciences, 06018 Ankara, Turkey; nesrin.turhan@sbu.edu.tr; 8 Faculty of Health Sciences, Antalya Bilim University, 07190 Antalya, Turkey; ismail.gomceli@antalya.edu.tr; 9 Department of Medical Biology, Acibadem Mehmet Ali Aydinlar University, 34752 Istanbul, Turkey

**Keywords:** gastric cancer, prognosis, biomarker, stroma

## Abstract

**Simple Summary:**

Gastric cancer is a leading cause of cancer-related death worldwide. Despite developments in the clinical management of this disease, currently, only 31% of patients with gastric cancer are expected to survive 5-years. However, not all patients follow the same course of the disease, and it is important to identify those individuals with potentially favorable or unfavorable outcomes to better define treatment options. For this purpose, we analyzed transcriptomic data from gastric tumors and identified 20 genes by which disease outcomes could be predicted. Unsupervised clustering of tumors based on the expression of these genes generated two major subgroups in a large number of cohorts. We show that patients with a poor prognosis have tumors with a more mesenchymal profile and a higher stromal content in both in silico and ex vivo experiments. We believe these findings will help shape the clinical management of gastric cancer.

**Abstract:**

Background: Molecular biomarkers that predict disease progression can help identify tumor subtypes and shape treatment plans. In this study, we aimed to identify robust biomarkers of prognosis in gastric cancer based on transcriptomic data obtained from primary gastric tumors. Methods: Microarray, RNA sequencing, and single-cell RNA sequencing-based gene expression data from gastric tumors were obtained from public databases. Freshly frozen gastric tumors (n = 42) and matched FFPE (formalin-fixed, paraffin-embedded) (n = 40) tissues from a Turkish gastric cancer cohort were used for quantitative real-time PCR and immunohistochemistry-based assessments of gene expression, respectively. Results: A novel list of 20 prognostic genes was identified and used for the classification of gastric tumors into two major tumor subgroups with differential stromal gene expression (“Stromal-UP” (SU) and “Stromal-DOWN” (SD)). The SU group had a more mesenchymal profile with an enrichment of extracellular matrix-related gene sets and a poor prognosis compared to the SD group. Expression of the genes within the signature correlated with the expression of mesenchymal markers ex vivo. A higher stromal content in FFPE tissues was associated with shorter overall survival. Conclusions: A stroma-rich, mesenchymal subgroup among gastric tumors identifies an unfavorable clinical outcome in all cohorts tested.

## 1. Introduction

Gastric cancer (GC) is the fifth cancer type with the highest incidence and the fourth most common cause of cancer-related death globally [1]. Although a variety of treatment options are available, the 5-year survival rate of gastric cancer patients in the United States is currently only 31% [2,3]. Surgical resection is considered curative; however, the majority of patients are diagnosed at later stages of the disease when the cancer has invaded other tissues, which hampers the opportunity for curative resection and results in a poor prognosis [4].

GC is a highly heterogeneous disease, so the discovery of prognostic and molecular subgroups among gastric tumors has been of interest, as even some of the most commonly used predictors, such as TNM staging, are constantly being revised; an example is the correct staging of N stage, which can be biased based on the number of harvested lymph nodes (eighth edition AJCC staging) [5]. Intestinal type and diffuse type GCs constitute two major histological classes, according to the Lauren classification. In 2019, WHO (World Health Organization) defined major histological subtypes of GC, which are papillary, tubular, mucinous, poorly cohesive, and mixed adenocarcinomas [6]. In parallel to the increase in the numbers of gastric tumors that are molecularly characterized at multi-omic levels, new studies in the literature reveal tumor subgroups that are biologically and/or prognostically distinct. For example, one such factor recently identified is the percentage of signet ring cells (SRC) in mixed-type GCs [7] and in poorly cohesive GCs [8]; in both cases, a percentage of SRCs above 10% have been associated with a better prognosis. In 2014, The Cancer Genome Atlas project defined a classification of gastric tumors into four subtypes: EBV (Epstein-Barr virus) positive, microsatellite unstable, genomically stable, and tumors with chromosomal instability; however, these subgroups did not show significant differences in terms of prognosis [9]. Although several prognostic biomarkers have been identified in gastric cancer, only conventional markers (CA19-9, CEA) and HER2 are currently used in clinical practice [10]. Various studies showed that increased CEA is a marker of poor prognosis in GC, although its independence from other confounding factors was found to be inconsistent in different cohorts [11,12,13]. Similarly, although elevated CA19-9 was associated with peritoneal metastases, stage, tumor depth, and nodal involvement [14], its sensitivity for prediction of recurrence is currently only 56% with a specificity of 74% [15]. Therefore, markers that can predict patient prognosis and molecular subtypes, which in turn may help develop better treatment options, are very much needed. 

A comprehensive evaluation of gene expression-based tests by AHRQ (the Agency for Healthcare Research and Quality) concluded that most of the prognostic/diagnostic tests available in the literature lack multivariate analysis (MVA) and thus analysis of independence from confounding factors [16]. Therefore, the practical value of these tests is unclear. In addition, AHRQ strongly recommends that prognostic tests be performed with multiple end-point measures of survival, such as overall survival, disease-free survival, cancer-specific survival, etc., and evaluated in multiple cohorts to avoid the identification of cohort-specific biomarkers. [16] Therefore, in this study, we aimed to identify prognostic biomarkers in GC that are independent of confounding factors when various end-point measures are utilized and are robust in multiple cohorts. Utilizing publicly available transcriptomic datasets, we obtained a list of 20 genes that consistently generated two major subgroups of tumors upon hierarchical clustering in multiple datasets with clearly distinct prognoses. Our data showed that this gene list could stratify both diffuse and intestinal-type tumors, was independent of clinical confounding factors, and was associated with both overall and disease-free survival. Further analysis showed that tumors from patients with poor prognosis had a dramatically higher stromal content, a more mesenchymal phenotype, and an enrichment of extracellular matrix-related gene sets.

## 2. Materials and Methods

### 2.1. Patient Characteristics and Clinical Samples

Forty-two patients above age 18 who were diagnosed with gastric cancer between 2008 and 2010 at Ankara Yuksek Ihtisas Hospital were included in the study. The NCCN (National Comprehensive Cancer Network) Gastric Cancer Clinical Practice Guideline was followed to perform surgery and lymphadenectomy in enrolled patients [17]. None of the patients received neoadjuvant therapy. Freshly frozen primary tumors (surgical specimens) were preserved at −80 °C. Forty of these patients with available paraffin blocks were included in immunohistochemistry. Clinicopathological data included overall survival (OS) time, follow-up status, age, gender, TNM stage, differentiation, perineural invasion, and lymphovascular invasion (Supplementary Table S1). Follow-up times ranged from 1 to 149 months. OS was calculated as the time from operation to death. Median survival was 15 months.

### 2.2. q-RT PCR

Primers for 5 prognostic genes (ACTA2, CALD1, HEYL, TAGLN, and TPM2), 2 reference genes (GAPDH, B2M), and epithelial and mesenchymal markers (CDH1, VIM) were designed using Primer-Blast (Supplementary Table S2) [18]. Tm values, GC contents, and amplicon lengths were set between 58 and 62 °C, 40 and 60%, and 70 and 140 bases, respectively. Total RNA was extracted from fresh frozen primary tumors using Trizol reagent (Thermo Fisher Scientific, Waltham, MA, USA) following the manufacturer’s protocol. RNA concentration and purity measurements were performed using Nanodrop One (Thermo Fisher Scientific). Of the RNA, 4000 ng was reverse transcribed into cDNA using the Revert-aid first strand cDNA synthesis kit (Thermo Fisher Scientific, #K1622) using random hexamer primers following the recommended protocol. cDNAs were then diluted to a 1:10 concentration for qRT-PCR experiments. A cDNA pool was prepared and used at 5 different concentrations (1:2, 1:4, 1:8, 1:16, 1:32). In order to assess the efficiency of primers, RT-PCR reactions with 5 µL iTaq Universal SYBR Green Supermix (Bio-Rad, Hercules, CA, USA, # 1725121), 0.6 µL forward and 0.6 µL reverse primers, 1.8 µL nuclease-free water, and 2 µL cDNA were run. Using an average cycle of threshold (Ct) and log of dilution concentration, a linear regression line was fitted, and primer efficiency was calculated with the formula: (10^(−1/the regression slope)^ − 1) × 100. Primers with an efficiency between 90 and 110% were used in further RT-PCR experiments. All RT-PCR reactions were run using iTaq Universal SYBR Green Supermix (Bio-Rad, #1725121) and the Light Cycler 480 II real-time PCR cycler (Roche, Basel, Switzerland). Ct values were normalized, and relative quantification was calculated using the delta-delta Ct method using the geometric mean of reference genes and one tumor sample as a reference [19]. 

### 2.3. TMA Construction

A tissue microarray (TMA), consisting of 40 tumors, was constructed, including one core (3 mm in diameter) for each tumor with the best representative morphology. FFPE, 4–5 μm thick tissue sections were stained with conventional H&E. The sections were also used for manual immunohistochemical (IHC) studies of HEYL, CALD1, TAGLN, and TPM2, and automated immunostaining of ACTA2, MLH1, PMS2, MSH2, and MSH6 antibodies listed in Supplementary Table S3.

### 2.4. Immunohistochemistry

Briefly, for manual IHC staining, the unstained slides obtained from TMA were deparaffinized at 60 °C and rehydrated gradually in a series of ethyl alcohols. Endogenous peroxidase activity was blocked with 3% H_2_O_2_ in methanol. Antigen retrieval was performed by microwaving in a citrate buffer (pH 6.0) solution. Sections were incubated with each primary antibody separately at the optimal dilution and titrated using the streptavidin–biotin peroxidase method for all antibodies. Sections were incubated with biotinylated goat anti-polyvalent secondary antibodies, and the avidin–biotin peroxidase method was then used. The signals were developed using the chromogen 3,3′-diamino benzidine (DAB), and finally, samples were counterstained with haematoxylin. The slides were dehydrated and prepared for microscopic examination. Automated immunostaining was performed via the Leica BOND-MAX IHC/ISH automated immunostainer (Leica Biosystems, Deer Park, IL, USA). TMA slides were also scanned with the Olympus VS 120 System and visualized via the OlyVIA software version 2.9.1 (Olympus, Hamburg, Germany). 

### 2.5. IHC Scoring and the Determination of MMR Status

Antigen expression for all antibodies except those used for mismatch repair mechanism proteins was assessed as follows: a simplified H score was determined by multiplying the percentage of positive cells with the intensity (scored from 0 to 3) of the staining. The sum of the H scores obtained from five genes was calculated. Two groups of tumors with summed H scores above and below the median were used for categorical statistical analysis. Additionally, percentages of tumor and stroma were evaluated separately for each tumor core on H&E slides.

Mismatch Repair (MMR) protein expression status was evaluated based on IHC stainings using MLH1, PMS2, MSH2, and MSH6 antibodies. Defective mismatch repair (dMMR) was defined as the loss of protein expression for at least one of the genes (MLH1, PMS2, MSH2, or MSH6). Proficient mismatch repair (pMMR) was defined as the presence of protein expression for all MLH1, PMS2, MSH2, and MSH6 proteins in the nuclei of neoplastic cells. Stainings that resulted in a non-decisive status for MMR protein expressions were classified as equivocal MMR (eqMMR), as described by Uner et al. [20]. For the eqMMR status determination, both protein expression levels and morphological evaluations were used.

### 2.6. Analysis of Transcriptomic Datasets

CEL files of GSE62254 [21], GSE15459 [22], GSE29272 [23], and GSE14208 [24] were RMA (Robust multiarray average) normalized using the “affy” package, and the “just.rma” function in R Bioconductor [25]. The “estimate” package was used based on Entrez gene IDs to generate stromal scores [26]. Stromal-high (ST-H) and -low (ST-L) designations were assigned using the median stromal score generated by ESTIMATE as the cut-off. Thirteen out of 20 probesets identified using GSE62254 and GSE15459 were available in GSE29272 and GSE14208 due to platform differences. GSE84437 [27], GSE13861 [28,29], GSE26899 [29], GSE26901 [29], and GSE28541 [29] datasets (Illumina platform) were quantile normalized using the “preprocessCore” package in R and then log-transformed. Annotations were performed according to the GPL file on the GEO page of each dataset. These datasets were clustered using probesets matching the gene names listed in Supplementary Table S4. For GSE26899 and GSE13861, 93 samples with the tumor type of gastric cancer and 65 gastric adenocarcinoma samples were included in the clustering analysis, respectively. For the GSE84437, GSE26901, and GSE28541 datasets, all samples were used. Clinical data for GSE13861, GSE26899, GSE26901, and GSE28541 were obtained from the supplementary files of Oh et al. [29]. Clinical data for other datasets were obtained from GEO (https://www.ncbi.nlm.nih.gov/geo/ (accessed on 23 April 2017: GSE62254, GSE15459 and GSE29272) (The rest of the datasets were accessed on October-November 2022)). TCGA Stomach adenocarcinoma (STAD) primary tumor RNA sequencing (RNAseq) data was downloaded from the GDC portal (https://portal.gdc.cancer.gov/ (accessed on 23 August 2022)) in STAR-counts format (n = 175). Genes with a count value of zero in less than 90% of the samples were used in further analysis. Counts were normalized using the “DEseq2” package and log transformed. Overall survival data were available for 316 patients. Days to death and days to last follow-up data were used as overall survival for patients with the status of dead and alive, respectively. TCGA-STAD subtype data was obtained from cbioportal (https://www.cbioportal.org/study/clinicalData?id=stad_tcga_pan_can_atlas_2018 (accessed on 16 September 2022)) in clinical data with the title “subtype”. Out of 20, 19 genes were available in this dataset, which was used to generate prognostic subgroups.

### 2.7. Determination of Genes Constituting the Prognostic Signature

Normalized log expression and overall survival data from GSE62254 (n = 300) and GSE15459 (n = 192) were analyzed by an in-house R script utilizing the “survival” library and “coxph” functions [30]. For each probeset, the Cox proportional hazards regression *p*-value and hazard ratio (HR) were obtained separately in each dataset. Probesets were then ranked based on Cox *p*-values from smallest to largest. Two rank values obtained from the two datasets were summed, generating a single ‘rank sum’ value for each probeset. Prognostic relationships with HR above 1 and below 1 were noted as associations with poor and good prognoses, respectively. Probesets associated with either a good or poor prognosis consistently in both datasets were included in further analysis. The top 20 probesets with the lowest rank sum were selected to constitute the gene signature (Supplementary Table S4).

### 2.8. Gene Set Enrichment and Ingenuity Network Analysis

The GSEA desktop application was downloaded from the Broad Institute (http://software.broadinstitute.org/gsea/downloads.jsp (accessed on 23 April 2017)). Gene sets were collapsed to gene symbols, and the maximum probe was selected for collapsing mode. The gene ontology “c5_all” was selected as the gene set database. Gene sets with less than 50 genes were not included in the analyses. Genesets with an FDR q value smaller than 0.25 were considered enriched. Protein network analysis was performed using the QIAGEN Ingenuity Pathway Analysis (QIAGEN IPA) (QIAGEN Inc., Redwood City, CA, USA, https://digitalinsights.qiagen.com/IPA (accessed on 3 January 2023)) application [31] based on differentially expressed probesets (absolute log fold change > 1, Benjamini–Hochberg corrected *p*-value < 0.05) between SU and SD groups in GSE62254.

### 2.9. Hierarchical Clustering Analysis

Cluster 3.0 and Treeview programs were downloaded (http://bonsai.hgc.jp/~mdehoon/software/cluster/software.htm (accessed on 23 April 2017)). Log expression values were standardized to a mean of zero and a standard deviation of 1 with the following formula: “(Value-Average)/Standard Deviation” for each gene or probeset prior to clustering analysis. Hierarchical clustering was performed by clustering both genes and arrays using Euclidian Distance as the similarity metric and Complete Linkage as the clustering method. Clustered data in “.cdt” format was then visualized with Treeview. SU and SD groups were categorized based on the major two branches of the dendrogram unless otherwise specified.

### 2.10. Clustering Based on EMT-Related Gene Expression

Probesets for the generic EMT signature of tumor tissues were provided by Tan et al. [32]. A list of probesets for 145 epithelial markers and 170 mesenchymal markers was used for the hierarchical clustering of gastric tumors in GSE62254 and GSSE15459. As epithelial and mesenchymal markers clustered in two major branches, samples with high expression of epithelial and low expression of mesenchymal markers were named “Epithelial” (E), while samples with the opposite profile of expression were named “Mesenchymal” (M). The rest of the samples with no clear pattern were named “Intermediate” (I).

### 2.11. Single-Cell RNA Sequencing Data Analysis

Single-cell RNA sequencing data from 26 gastric tumors were obtained from GSE183904. The count data was already filtered as described by Kumar et al. [33]. Using the “Seurat” package in R Bioconductor (Version 4.1.1), counts were log-normalized using the scale of 10,000 [34]. 2000 variable features were determined using the “FindVariableFeatures” function. Data were scaled with the “vars to regress” option set to mitochondrial percentage to regress out unwanted sources of variation. Principal component analysis was performed using the “RunPCA” function. Cell clusters were defined using the “FindNeighbors” and “FindClusters” functions. For non-linear dimension reduction and visualization of the clusters, the “RunUMAP” function was utilized. Cell clusters were identified based on markers listed in Che et al. [35], with minor changes. We added the NKG7 gene for the identification of natural killer (NK) cells. The cell cluster expressing CD68, CD163, CD14, and LYZ was named “Myeloid cells”. Out of 20, 18 prognostic genes were available in this dataset.

### 2.12. Statistical Analysis

Kaplan–Meier graphs were generated using Graphpad Prism 8 (Graphpad Prism 8 Software, San Diego, CA, USA). Spearman correlation, chi-square and Fisher’s exact tests, and multivariate Cox regression analysis were performed using IBM SPSS Statistics for Windows Version 23 (IBM Corp., released 2015; Armonk, NY, USA). Patients with nonzero survival data (OS or RFS–recurrence-free survival, DFS–disease-free survival) and available status information were included in these analyses. Documented recurrence (0 = no, 1 = yes) was used as the status for DFS. Log-rank multiple cut-off graphs were generated as described previously [36]. 

## 3. Results

### 3.1. Identification of Prognostic Markers in Gastric Cancer

**Figure 1:**
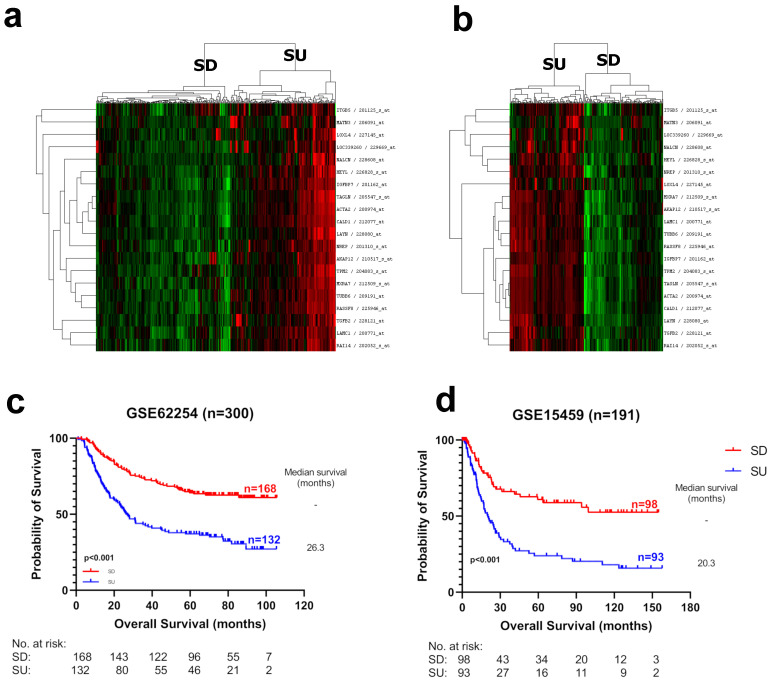
Hierarchical clustering analysis of gastric cancer tumors with prognostic genes. Twenty probesets that were significantly associated with survival data were used for hierarchical clustering in discovery cohorts: (**a**) GSE62254 (n = 300) and (**b**) GSE15459 (n = 192). Red, black and green colors indicate high, intermediate and low expression, respectively. Two groups with discernible high and low expressions for all 20 probesets were labeled as “SU” and “SD” groups, respectively. Kaplan–Meier plots are shown for patients with the SU/SD label in (**c**) GSE62254 and (**d**) GSE15459. Log-rank *p*-values are indicated.

**Figure 2:**
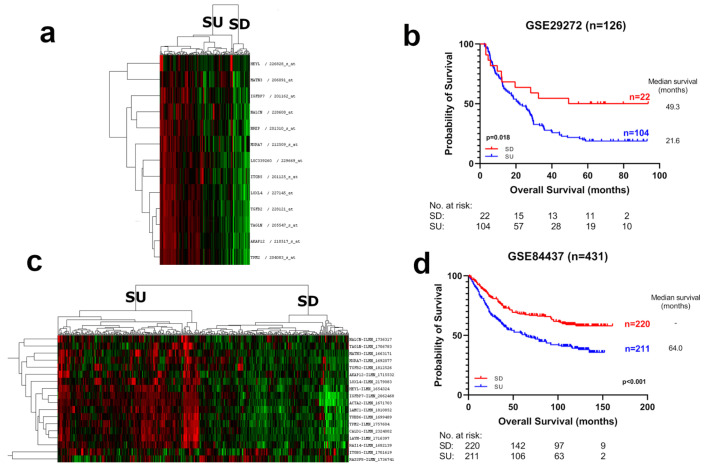
Hierarchical clustering analysis and Kaplan–Meier graphs of gastric cancer tumors with prognostic genes in validation cohorts. (**a**) Out of 20, 13 prognostic probesets that were available on the HGU133A platform were used for hierarchical clustering in GSE29272, and (**c**) Eighteen Illumina probesets of prognostic genes were used for GSE84437. Red, black and green colors indicate high, intermediate and low expression, respectively. Two major branches with low and high overall expression were assigned to the SU and SD groups. Kaplan–Meier graphs and log-rank *p*-values are shown for (**b**) GSE29272 and (**d**) GSE84437.

In order to identify RNA-based biomarkers for gastric cancer prognostication, we performed Cox regression analysis predicting overall survival for all probesets in two discovery datasets, GSE62254 (n = 300) and GSE15459 (n = 192). Rank values based on *p*-values obtained from the two datasets for each probeset were summed, and the top 20 probesets with the lowest rank numbers were selected for the prognostic panel (see Section 2.7) (Supplementary Table S4). Expression of all genes within the list was associated with poor OS. Hierarchical clustering analysis using the 20 probesets identified two clearly distinct tumor groups with noticeable high and low expressions in both discovery datasets. As all these genes were related to increased stroma in tumors (see below), they were named “Stromal-UP” (SU) and “Stromal-DOWN” (SD), respectively (Figure 1). The SU group had a significantly worse prognosis compared to the SD group, in both datasets (GSE62254; HR: 2.47, *p* < 0.0001. GSE15459; HR: 2.66, *p* < 0.0001) (Figure 1). The median survival of the SU groups was 26.3 and 20.3 months, whereas the median survival of the SD groups was longer than the follow-up time: 120 and 200 months in the GSE62254 and GSE15459 datasets, respectively, indicating a dramatic difference in clinical outcome. SU/SD distinctions were robustly observed upon clustering in independent gastric tumor datasets (GSE29272, GSE84437), and patients with SU tumors showed poor prognoses compared to the SD group in these datasets as well (Figure 2). Clustering analysis with 19 of the 20 genes available in The Cancer Genome Atlas (TCGA) stomach adenocarcinoma (STAD) RNAseq data resulted in three major subgroups with high (SU), intermediate (SI), and low expression (SD), all of which had significantly different prognosis (*p* = 0.011) (Supplementary Figure S1). In this dataset, the SD group was distinctly separated from the SI and SU groups based on both the expression and prognostic profiles (Supplementary Figure S1). These findings show that SU–SD classification is a strong predictor of prognosis in all cohorts analyzed.

According to the American Agency for Healthcare Research and Quality, studies of prognostic biomarkers should be assessed using multiple clinical outcome types [16]. Therefore, we re-analyzed our prognostic signature using DFS as a clinical outcome in GSE62254. Indeed, the SU group was significantly associated with shorter DFS (Supplementary Figure S2). 

We further applied this classification to three other datasets that had both OS and RFS available (Supplementary Figure S3). The SU and SD groups identified significantly different prognostic subgroups in GSE26901 as well (Supplementary Figure S3). In datasets with smaller sample sizes (GSE26899, GSE13861), the trend remained the same as SD groups had better median survival, although the differences were not significant (Supplementary Figure S3). In order to better understand the potential underlying reasons for the smaller differences in prognosis among SU–SD patients in these cohorts, we evaluated whether differences in treatment protocols among these patients had an effect. The regimens overlapped between datasets: standard adjuvant chemotherapy (either single-agent 5-fluorouracil or a combination of 5-fluorouracil and cisplatin/oxaliplatin, doxorubicin, or paclitaxel) [29] was given, while some patients received no treatment. When the prognosis of patients with and without adjuvant chemotherapy treatment was compared, we noted that patients who received therapy showed a significant survival benefit in GSE26899 but not in GSE26901 or GSE13861 (Supplementary Figure S4). In addition, we noted that 79.6%, 74.6%, and 56.4% of the patients who received treatment had an epithelial phenotype (EP as defined by Oh et al.) [29] in GSE13861, GSE26899, and GSE26901, respectively. Therefore, a higher percentage of EP tumors in the treatment group for GSE26899 may be related to a higher treatment benefit compared to non-treated patients. This was not observed for GSE13861, likely due to the availability of only 16 patients with no treatment. Considering the dramatic prognostic difference between SU and SD groups in GSE26901, but not in GSE26899 (Supplementary Figure S3E–I), our data suggest that SU–SD classification is an indicator of the intrinsic biological potential of the tumor to recur and progress, which can, however, be affected by treatment. Further stratification of these patients based on treatment showed that in GSE26901, the prognostic difference between the SU and SD groups was less in treated patients compared to the untreated group (*p* > 0.1, *p*-value: 0.023, respectively) (Supplementary Figure S5). In GSE26899, no significant survival difference was noted between SU and SD in any of the treatment groups (Supplementary Figure S5). To evaluate whether the SU–SD signature could be a predictor of treatment response, we compared the prognosis of patients with and without treatment within the SU and SD groups. For GSE26899, patients with either SU or SD tumors benefited from therapy, whereas in GSE26901, neither group benefited. Therefore, our data suggest that the SU–SD classification is not a predictor of treatment response (Supplementary Figure S6) and that it is a predictor of overall survival independent of treatment (Supplementary Table S5).

Up until now, we compared SU–SD groups based on clear patterns in hierarchical clustering analysis. However, we also encountered two datasets, GSE14208 and GSE28541, for which this pattern was less clear compared to the datasets previously analyzed (Supplementary Figure S7). In GSE14208, which included samples obtained from metastatic gastric cancer patients, there was no difference between the overall survival of the SU and SD groups. In GSE28541, the heatmap showed correlating gene expression patterns for a subgroup of genes, unlike other datasets where we observed correlating expression patterns for all genes (Supplementary Figure S7). Despite that, we validated the trend at borderline significance (*p*-value: 0.06). Overall, the SU–SD signature was a predictor of prognosis in gastric cancer, as was evident, especially in cohorts with larger sample sizes, but it was not a predictor of treatment response.

### 3.2. Biological Characteristics of Prognostic Groups

In order to illuminate the biological differences between SU and SD tumors, we performed GSEA (gene set enrichment analysis; see Section 2.8). Zero and three genesets were enriched in SD samples, whereas 63 and 188 genesets were enriched in SU samples in GSE15459 and GSE62254, respectively. These results suggested a more heterogeneous molecular profile in SD tumors compared to the SU group. For the genesets that were enriched in the SU group, we noted common patterns for the two datasets related to the extracellular matrix, actin cytoskeleton and binding, wound healing, and negative regulation of cell cycle and metabolism (Supplementary Table S6, Supplementary Figure S8). These findings showed that the patient group with the worse prognostic profile (SU) may have less proliferation but more extracellular matrix (ECM) involvement and cell migration, suggesting a more mesenchymal phenotype when compared to patients with a relatively better prognostic profile (SD). Therefore, we utilized a published gene list of EMT markers [32] and classified gastric tumors into epithelial (E), intermediate (I), and mesenchymal (M) (Supplementary Figure S9; see Section 2.10). The distribution of SU–SD samples was significantly related to the E–I–M profiles (*p* < 0.00001 for both datasets) (Supplementary Table S7). The majority of the SD tumors were epithelial (GSE62254), and most of the SU samples were mesenchymal (GSE15459). When only E and M samples were considered, the E profile overlapped with SD, and the M profile overlapped with the SU group to a large extent. The I group did not have a consistent overlap with either prognostic subgroup. As enrichment of ECM-related gene sets suggests a more stromal profile for the SU group, we utilized a previously defined algorithm, ESTIMATE, which uses gene expression signatures to infer the fraction of stromal and immune cells in tumor samples [26]. In both GSE62254 and GSE15459, SU groups had significantly higher stromal scores, as calculated via ESTIMATE (Supplementary Figure S10). To evaluate the molecular interactions that are differentially regulated in the SU group, we performed an Ingenuity network analysis based on differentially expressed genes between the SU and SD groups in GSE62254 (see Section 2.8) (Supplementary Figure S11). The summary graph generated by Ingenuity, which highlights the major biological themes in our analysis, had TGF-beta as a hub, with several key mesenchymal markers (TWIST1, FN1, TWIST2), the stem cell marker CD44, HIF1A (the master regulator of cellular and systemic homeostatic response to hypoxia), and inflammatory markers (IL1-alpha and IKBKB) (Supplementary Figure S11A). In addition, the activation of multiple cellular processes related to fibrosis and cellular movement, including the migration of carcinoma cells, was predicted. We noted molecular interactions between FBN1 (an extracellular matrix glycoprotein fibrillin), multiple collagens (COLA1, COL3A1, COL1A2, COL6A1, COL6A3), TPM2 (an actin filament binding protein), PDLIM3 (an actinin-associated protein), junction proteins (AMOTL1, GJC1), TIMP3 (a metalloproteinase inhibitor), and AMIGO2 (involved in cell adhesion), supporting a denser stroma for the SU group. In the top network with the highest number of focus molecules, the activation of NF-kB was predicted (Supplementary Figure S11B) in relation to the induction of transcription of extracellular matrix remodeling enzymes, influencing the expression of VEGF and E-cadherin, promoting EMT, angiogenesis, and metastasis [37]. Overall, these findings indicate that the SU group has more stromal involvement, less proliferative activity, and a more mesenchymal phenotype, all of which are likely due to the activation of TGF-beta and NF-kB, which are also associated with a more cancerous stem cell-like environment. The SD group was comprised of rather more epithelial and less aggressive tumors.

### 3.3. Expression of Prognostic Genes in Gastric Tumors at the Single-Cell Level

As GSEA and network analysis results contained consistent enrichment of ECM-related components in the SU group, in which the prognostic genes were expressed at high levels, we next asked whether these genes are expressed in cancer cells or the stromal cells of the tumor microenvironment (TME). Analysis of a single-cell RNA sequencing dataset that included 26 primary gastric tumors (GSE183904) showed that six of the genes (IGFBP7, CALD1, TAGLN, TUBB6, LAMC1, and RAI14) were expressed mainly in cancer-associated fibroblasts (CAFs) and endothelial cells (Supplementary Figure S12), while ITGB5, MXRA7, ACTA2, and TPM2 were expressed primarily in CAFs (Supplementary Figure S12). One gene, AKAP12, was expressed in both CAFs and mast cells. There were 7 genes (MATN3, LAYN, TGFB2, LOXL4, RASSF8, HEYL, and NALCN) for which very low or no expression was noted in the cell types detected in the dataset. Strikingly, none of the genes were expressed in epithelial cells, except for very weak expression from ITGFB5 and TPM2. These data together suggest that the SU–SD signature defines an expression profile mostly originating from the stromal cells in the TME that is strongly associated with clinical outcomes.

### 3.4. Evaluation of the SU–SD Signature with Clinical and Biological Subgroups

TCGA has classified gastric tumors into four main subtypes: tumors positive for Epstein-Barr virus (EBV), microsatellite unstable tumors (MSI), genomically stable tumors (GS), and tumors with chromosomal instability (CIN) [9]. The distribution of these subtypes was related to subgroups defined by the SU–SD gene signature within TCGA stomach tumors (high (SU), low (SD), and intermediate (SI) (*p* < 0.001) (Supplementary Table S8). We noted that GS-type tumors, which are enriched for the diffuse histological variant and mutations of *RHOA* or fusions involving RHO-family GTPase-activating proteins, contained the highest percentage of SU samples (60%) (Supplementary Table S8). In contrast, the largest percentage of SD samples were observed in EBV and MSI types. EBV tumors are known to display recurrent *PIK3CA* mutations, extreme DNA hypermethylation, and amplification of *JAK2*, *PD-L1,* and *PD-L2*, whereas the MSI profile is associated with elevated mutation rates in many genes. Both types have been defined as responsive to immunotherapy [38]. None of the TCGA subtypes overlapped more than 60% with SU–SD groups, indicating that the SU–SD classification is related but biologically distinct from the previously defined molecular subtypes. Indeed, multivariate analysis showed that the SU–SD signature was an independent prognostic predictor when TCGA subtypes were included in the model (Supplementary Table S9). In this line, the SU–SD signature could stratify CIN and MSI types, suggesting that the involvement of ECM and stroma-related biological patterns is prognostically relevant within these groups (Supplementary Figure S13). Stratification within GS and EBV subtypes is inconclusive, likely due to the very low sample size (Supplementary Figure S13). 

### 3.5. SU–SD-Based Classification Is an Independent Prognostic Marker

**Figure 3:**
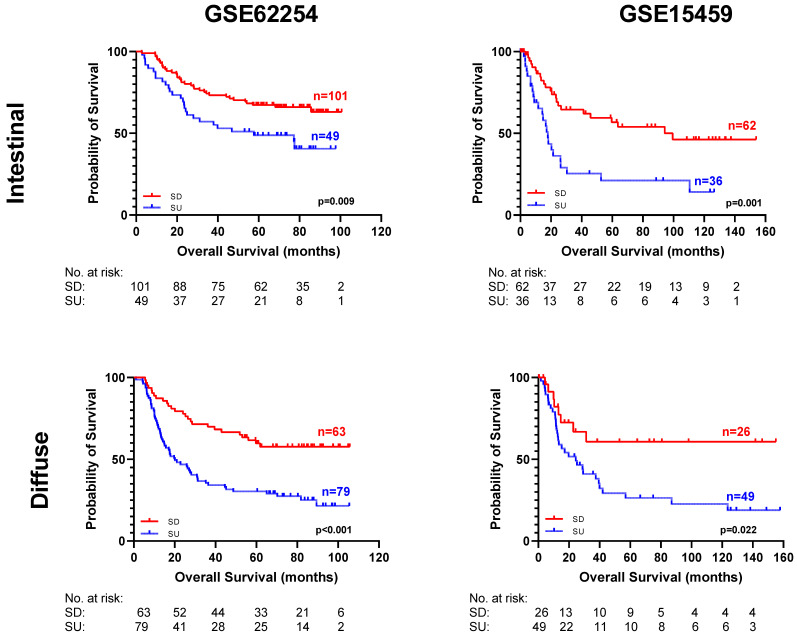
Kaplan–Meier plots for SU–SD groups within intestinal and diffuse type tumors in GSE62254 and GSE15459. Log-rank *p*-values are shown.

Multivariate analysis showed that the SU–SD classification was independent of clinical confounding factors: age, tumor pathological subtype (diffuse, intestinal, mixed), location, stage, H. pylori infection, MLH1 status, and previously defined molecular subtypes [21], among others (Supplementary Table S10 and Table S11). In this model, we found SU–SD was one of the three sole prognostic factors, together with stage and tumor subtype. Thus, as expected, our prognostic signature stratified patients with either intestinal or diffuse-type tumors into subgroups with significantly different clinical outcomes in terms of both OS and DFS (Figure 3; Supplementary Figure S14). Similarly, among four distinct gastric tumor subgroups previously defined by the Asian Cancer Research Group (ACRG), MSI, MSS/EMT, MSS/TP53^+^ (MSS with intact TP53 activity), and MSS/TP53^-^ (MSS with TP53 functional loss), MSI- and MSS/TP53^-^-type tumors could be stratified into significantly different prognostic groups based on SU–SD classification (Supplementary Figure S15). Although we observed a relatively worse prognosis in the SU group compared to SD patients in MSS/TP53^+^, this was not significant (*p* = 0.09). Univariate analysis of common clinical confounding factors in GSE15459 and GSE29272 showed that stage was a significant prognostic factor in GSE15459 but not age, gender, or pathological subtypes, whereas none of them were significant in GSE29272 (Supplementary Table S12). When stage and SU–SD classification were included in a multivariate model in GSE15459, SU–SD classification was an independent prognostic factor, confirming our findings in GSE62254 (Supplementary Table S13). We then analyzed the prognostic relationship of the SU–SD signature within each stage. The SU–SD signature was able to significantly stratify stage II and stage III tumors but not stage I and IV tumors (Supplementary Figure S16). As the SU–SD classification was related to the presence of stroma within the tumor, we tested whether stroma itself was a prognostic factor and, if so, whether SU–SD was a prognostic factor independent of stromal presence. For this purpose, we used the median stromal score, as generated by ESTIMATE, to stratify patients into stromal-high (ST-H) and -low (ST-L) groups. Univariate analyses showed that the ST-H phenotype was associated with worse overall survival in GSE62254 (*p*-value: 0.044, H.R.: 1.39), but not in GSE15459 (*p* = 0.1, H.R. = 1.41). We next performed MVA with both stromal score-based and SU–SD classifications included in the model in GSE62254. This showed that SU–SD was associated with survival, independent of stromal classification; Cox *p*-values were <0.001 and 0.209, H.R.s: 2.86 and 1.28, for SU–SD and stromal classification, respectively. Overall, our data indicated that the SU–SD signature was an independent prognostic classifier and that it was able to prognosticate especially MSI- and MSS/TP53^-^-type tumors and those of stages II and III.

### 3.6. Validation of the SU–SD Signature Ex Vivo

**Figure 4:**
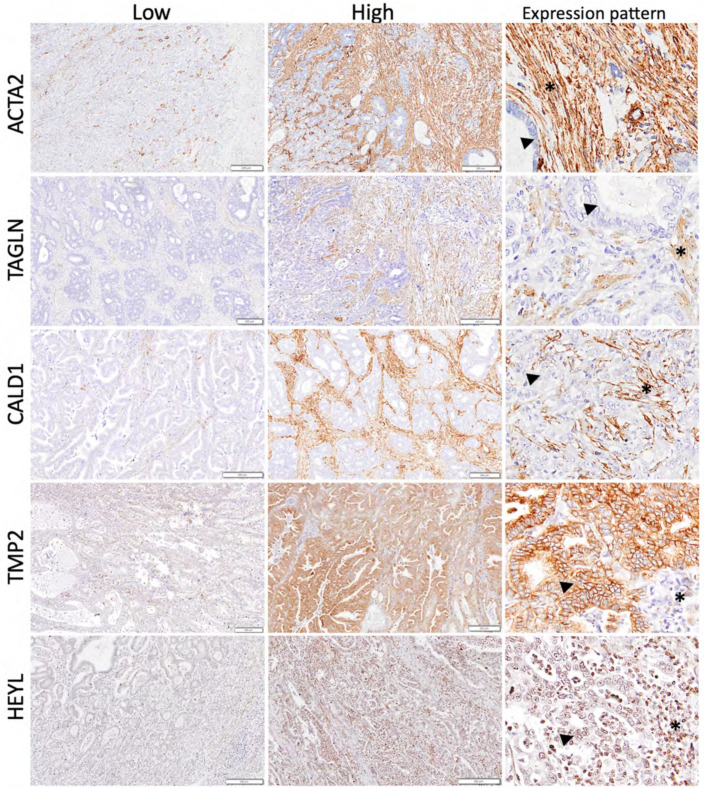
IHC-based staining of protein levels in tissue (representative low and high areas, ×40 magnification) and protein expression patterns of the proteins regarding the five genes (×200 magnification). ACTA2, TAGLN, and CALD1: cytoplasmic staining in spindle-shaped stromal cells, mostly regarding fibroblasts (asterisk) and negative in neoplastic epithelial glands (arrowhead). Staining intensity was stronger in ACTA2, while TAGLN and CALD1 showed weaker staining. TMP2: Moderate to strong cytoplasmic and membranous staining in neoplastic epithelial glands (arrowhead). Additionally, stromal cells showed positivity (an asterisk) in various degrees. HEYL: weak to moderate cytoplasmic and nuclear staining in neoplastic epithelial glands (arrowhead) with moderate expression in stromal cells, including inflammatory cells (asterisk).

To validate the SU–SD signature ex vivo, we selected five genes (HEYL, ACTA2, TPM2, CALD1, and TAGLN) that are expressed at the protein level in gastric tumors as defined in the Human Protein Atlas (https://www.proteinatlas.org/ (accessed on 10 January 2019)). Four of the genes, ACTA2, TPM2, CALD1, and TAGLN, were known to be involved in muscle contraction and differentiation and the actin cytoskeleton in cell motility, suggesting a consistent biological pattern with stromal features associated with the poor prognostic group [39,40,41,42,43]. We performed IHC using FFPE tissues from Turkish gastric cancer patients (n = 40), 12 of which were classified as gastric carcinoma with lymphoid stroma based on morphological evaluation [6] (Supplementary Figure S17) (Supplementary Table S1). At the time of diagnosis, 26 and 16 patients were above and below 60 years of age, respectively. Twenty-eight patients were male and 14 were female. Seven out of 42 patients were censored at the time of the last follow-up. IHC-based staining of protein levels in tissue showed different patterns regarding the five genes. Protein expression patterns of ACTA2, TAGLN, and CALD1 were similar, and the expression of ACTA2 was the strongest among these. These three proteins revealed cytoplasmic staining in spindle-shaped stromal cells, mostly fibroblasts, and were negative in neoplastic epithelial glands. TMP2 showed moderate to strong cytoplasmic and membranous staining in neoplastic epithelial glands. Stromal cells were also positive to various degrees. HEYL revealed weak to moderate cytoplasmic and nuclear staining in neoplastic epithelial glands with moderate expression in stromal cells, including inflammatory cells (Figure 4).

Stromal expression of ACTA2 was positively correlated with stromal expression of TAGLN and CALD1 (rho > 0.35, *p* < 0.05, n = 40) (Supplementary Table S14). Both neoplastic and stromal expression of TPM2 was positively correlated with the expression of HEYL in all cells evaluated: neoplastic, stromal, and inflammatory cells (rho > 0.35, *p* < 0.05, n = 40) (Supplementary Table S14). The staining patterns were scored based on both intensity and percentage of cells expressing the genes (simplified H score, see Section 2.5), separately for neoplastic cells, stromal cells, and inflammatory cells (only for HEYL). High stromal expression of TAGLN was associated with poor overall survival in this cohort (*p* = 0.041) (Supplementary Figure S18C), whereas prognostic relationships for HEYL, ACTA2, TPM2, and CALD1 could not be validated. To evaluate the total expression levels, the sum of the simplified H scores from the five genes was calculated. The median cut-off was used to classify tumors with high and low expression, which was associated with MMR status, gender, and perineural invasion (Fisher’s exact test *p*-values: 0.022, 0.048, and 0.019, respectively). Overall, a higher expression value overlapped with a higher percentage of perineural invasion, MMR-proficient tumors, and male patients.

As the SU–SD gene signature stratified tumor groups that differed highly in their stromal content, we next evaluated the percent stromal ratio in these tumors. A high stromal ratio was significantly associated with an unfavorable prognosis in line with the in silico findings (see Section 3.5) (Supplementary Figure S18A,B). 

In addition to protein-level expression, we also quantified the expression of these five genes via qRT-PCR in matched, freshly frozen primary tumor samples of the same cohort (n = 42). Expression of the five genes is strongly inter-correlated, confirming in silico findings (average rho: 0.75) (Supplementary Table S15). Expression of all five genes showed a strong positive correlation with the mesenchymal marker VIM (rho > 0.6, *p* < 0.05) and a negative correlation with the epithelial marker CDH1 (rho < −0.4 and *p* < 0.05 except for HEYL: rho:−0.3; *p*-value: not significant). Analysis of paired samples with both FFPE tissues and fresh frozen tumors showed that transcript levels of ACTA2, TPM2, CALD1, and TAGLN were significantly and positively correlated with the stromal percentage of the tumor. However, in this cohort, qRT-PCR-based expression of none of the genes was significantly associated with clinical outcome (Cox *p*-values >0.05). 

Overall, this data shows that the concordant expression of five of the 20 genes in the SU–SD signature is correlated with the stromal presence, which is a significant predictor of prognosis. 

## 4. Discussion

Although therapy options have improved in GC, prognosis of these patients are still quite poor, especially when diagnosed at later stages. Understanding the dynamic biology of disease subtypes that are associated with differential clinical outcomes is crucial to pave the way to alternative and more potent treatment options. Increasing number of high-throughput datasets that are publicly available, enables identification of novel biomarkers that are applicable to more cohorts and/or more specific subtypes. In this study, we defined a novel list of genes that can generate gastric cancer subgroups with dramatically different prognosis. Clustering-based classification of tumors based on this gene list validated prognostic association in multiple datasets in terms of both OS and DFS. We showed that SU group had higher ECM and tumor stroma-related gene expression. It can prognosticate MSS and TP53^-^ and MSI types among ACRG subtypes but not EMT group, as SU profile overlapped with mesenchymal gene expression. Therefore, we identified SU group as an aggressive, stroma-rich, mesenchymal subtype. In line with these findings, other studies showed that a mesenchymal profile was associated with poor prognosis in gastric cancer [29]. ECM-related components were also implicated in gastric cancer, multiple collagens and cancer associated fibroblast markers were identified among upregulated genes in tumor compared to normal [44]. Here we show that they are indeed key determinants of tumor progression independent of clinical confounding factors.

IHC-based stainings for the protein levels of the five genes revealed that three out of five genes (CALD1, TAGLN and ACTA2) were expressed exclusively from the stroma. For the other two genes (TPM2 and HEYL), in addition to stromal expression, mild to moderate expression was observed in neoplastic cells. IHC-based expression of four genes and RNA level expression of five of the prognostic genes, when quantified in our ex vivo cohort, were not associated with clinical outcome. One possible reason for this could be ethnicity-related molecular differences between the ex vivo cohort of Turkish patients and in silico cohorts, which included mostly patients from East Asia, where the incidence/mortality ratio is higher than that in Turkey [45]. When GSE62254 and our cohort were compared, the median survival times were 77 and 15 months, respectively although the clinical parameters of the two cohorts were similar (age, stage, gender), indicating different innate prognoses of these cohorts. As the sample size of the ex vivo cohort was much smaller compared to the in silico datasets, it is likely that the prognostic associations may be improved in studies with larger cohorts. We showed that these markers are associated with EMT and are expressed predominantly from tumor stroma, however a significant number of tumors (12/40) in our cohort had a lymphocyte-rich stroma rather than a desmoplastic one. Therefore, a dramatically higher percentage of lymphoid rich tumors (30%) in this cohort compared to WHO statistics (1–7%) [6], might also have influenced the validation of markers that are expressed primarily from tumor stroma. 

It is known that gastric carcinomas with lymphoid stroma have a better prognosis and they benefit from different chemotherapy models (eg. 5-FU is contraindicated and they are more sensitive to irinotecan etc.) [6]. In addition, this group of tumors benefits from immunotherapies such as anti-PDL-1 and anti-PD-1 [46]. However, unexpectedly, the overall survival in our ex vivo cohort was worse than the in silico cohorts. We may speculate that the main reason for this discrepancy is that most of our patients were diagnosed before identification and inclusion of the lymphoid-rich group in guidelines and thus specific treatment models were not available for those patients yet. Most of them were not treated with currently available chemotherapy options and immunotherapies.

Our results showed that SU–SD signature is not a predictor of treatment response. In cohorts with a higher benefit from treatment which was likely to improve survival of poor survivors, SU–SD group could not stratify patients significantly. In line with this, the prognostic difference was dramatic in untreated patients, suggesting that this signature indicates an intrinsic biological phenotype of the pre-treatment tumor, which can then be changed by treatment. A better prognostic separation in datasets with a higher percentage of patients who did not receive treatment (GSE26901: 63.9% vs GSE26899: 27.9%) confirmed these findings. This signature was a good prognostic predictor in stage II and III disease, but not in I and IV which is further supported by the poor performance of this classification in metastatic gastric cancer patients. In line with this observation, analysis of the GSE28541 dataset, consisting mostly of stage IV tumors (52%), did not reveal a clear SU–SD pattern in clustering and had a prognostic difference with a borderline significance (0.06). Therefore; the stromal and mesenchymal phenotype that predicts recurrence, does not seem to be a main prognostic indicator in already metastasized and late stage disease.

An analysis of the molecular subtypes defined by the TCGA consortium showed that the SU–SD signature was independent of these subtypes and that the highest percentage of the patients from both the EBV and MSI subgroups were in the SD group. Both the EBV and MSI type tumors are considered as eligible for immunotherapy [38]. It has been shown that the PD-L1/PD-1 pathway is activated and could be considered an emerging therapeutic target, particularly for EBV and MSI GCs [47]. Therefore, the SD group may more likely benefit from immunotherapy. As EBV and MSI types were shown to have a good prognosis previously [48,49], an overlap between these types and the SD group may be expected, as the SD group also had a favorable clinical outcome in almost all cohorts we tested. As other immune markers, such as lower CD26 levels associated with an immune defective antitumor response [50] are defined they can be analyzed in further studies. Altogether, we speculate that the crosstalk between immune cells and cancer cells in the tumor microenvironment may also differ between the subgroups in this study. We observed an intermediate group in the TCGA-STAD dataset that was not present in microarray datasets, likely due to the wider range of expression obtained by RNAseq. In addition, all four TCGA subtypes had tumors distributed across SU–SI–SD groups indicating that both classifications are likely to contribute to the molecular subgrouping of gastric cancer.

Analysis of stromal scores in SU–SD groups showed that, SU tumors had dramatically high stromal content compared to SD tumors. A transcription factor, HEYL (Hes Related Family BHLH Transcription Factor With YRPW) included in our list was suggested previously as a potential prognostic marker for gastric cancer [51]. Another study showed that HEYL expression correlated with the stromal transformation of the TME [52]. Upon its overexpression, transcriptional activation of cadherin 11 (CDH11) was observed [52], which was linked with stroma–epithelium interaction [53]. Another gene in the SU–SD signature ACTA2 (alpha)-SMA is a specific marker for myofibroblasts and is a hallmark of myofibroblast differentiation [54,55]. It is expressed mainly by transforming growth factor-β (TGF-β)-dependent myofibroblastic CAFs [56]. ACTA2 was included in a 32-gene signature that defined four molecular and prognostic subgroups of gastric cancer [57]. In this study, ACTA2 expression was associated with the mesenchymal group with TGFB activation which did not benefit from the 5-FU + Platinum. 87% of these patients were not responsive to immunotherapy. In parallel to the association with TGFB pathway activation observed in this study, our list included TGFB2 which is considered to be a link between epithelial–mesenchymal transition (EMT) and tumor mutation burden in gastric cancer, and its high expression was associated with poor prognosis in multiple datasets [58]. TGFB2 was included in a four gene signature (FN1, TGFB2, TGFBR2, and TGFBI), as an indicator of CAF abundance, which predicted survival in TCGA pan-cancer cohort comprising 9356 patients from 32 cancer subtypes [59]. In parallel, TGFB2 secreted by CAFs was found as being essential for maintaining stemness of colorectal tumorspheres [60]. A previous study suggested that another gene listed in our panel, NREP, was associated with poor prognosis and may be involved in the activation of cancer-associated fibroblasts and the epithelial–mesenchymal transition, with transforming growth factor β1 mediating both processes [61]. IGFBP7 was identified as a tumor stroma marker of epithelial cancers, and stromal expression of IGFBP7 was an indicator of poor survival in colorectal cancer [62,63]. When SW480 colorectal cancer cells were co-cultured with fibroblasts, IGFBP7 expression was induced in fibroblasts at both the mRNA and protein levels [64]. In turn, IGFBP7-expressing CAFs can induce colony formation in colon cancer cells suggesting a paracrine tumor–stroma interaction [63]. Furthermore, when expressed by tumor cells IGFPB7 can promote anchorage-independent growth [63]. CALD1 gene was exclusively expressed in stromal cells in colorectal adenocarcinoma, and elevated expression of CALD1 and IGFPB7 in stromal cells predicted robustly shorter disease-free intervals [65]. In addition, the levels of these proteins (CALD1, IGFBP7) were upregulated by TGF-β in colon fibroblasts [65]. Therefore, high expression of these genes pointed out a mesenchymal, CAF abundant, and TGFB pathway active phenotype associated with disease progression.

Several genes in our panel are implicated in the ECM and tumor stroma-related mechanisms. Expression of ITGB5 in gastric tumors is associated with extracellular matrix organization, focal adhesion and ECM-receptor interaction based on gene functional enrichment and KEGG pathway analysis [66]. Matrix remodeling associated 7 (MXRA7) was identified via its coexpression with genes known to mediate cellular adhesion or extracellular matrix remodeling [67]. A limited number of studies were conducted regarding the function of this gene so far, and our study suggests that MXRA7 is expressed in stroma-high gastric tumors and may be also involved in remodeling of cancer-associated TME. Yu et al. showed that TAGLN, an actin binding protein, was upregulated in CAFs and through which CAFs enhanced tumor metastasis in vitro and in vivo [68]. TAGLN gene was also included in an autophagy-stroma gene signature which was an independent prognostic factor for colorectal cancer [69]. Furthermore, overexpression of TAGLN was strictly localized to the tumor-induced reactive myofibroblastic stromal tissue compartment in lung adenocarcinoma suggesting TAGLN as a marker of active stromal remodeling in the vicinity of invasive carcinomas [70]. TAGLN and TPM2 genes were among five genes identified as fibroblast-specific biomarkers of poorer prognosis of CRC [71]. AKAP12 was among the genes that are potentially involved in tumor–stroma talk in pancreatic cancer [72]. LAMC1 was included in a four-gene model for prognostic risk prediction as a stromal gene [73], as it is involved in ECM-receptor interaction pathway (KEGG pathways: hsa04512). In keratocystic odontogenic tumors, LOXL4 gene was positively correlated with stromal microvessel density (r = 0.882) suggesting an involvement in enhancement of angiogenesis [74]. Overall, itis highly likely that expression of the above mentioned genes detected in bulk tumors are primarily derived from the tumor stroma rather than epithelium also as supported by single-cell RNAseq data in this study. Expression of several genes (MATN3, NALCN, RASSF8, LOXL4) were minimal or not detected in this single-cell RNAseq dataset, which could be due to small sample number, lower stromal content in those tumors and inter-tumoral heterogeneity. In line with the previous data indicating positive correlation of stromal content with HIF1A expression in many solid tumors [75], we found HIF1A gene among the differentially expressed genes in the SU group. Expression of this gene is a mark of increased hypoxia which promotes EMT, and thus migration and invasion of gastric cancer cells [76]. Network analysis revealed subnetworks of collagens, ECM components, mesenchymal markers, and predicted a higher activity of fibrosis and cancer cell movement related biological processes supporting our previous findings. Thus, our data pointed out a subgroup of gastric cancers that express genes involved in the ECM and tumor stroma related processes and may also have a hypoxic microenvironment in gastric cancer.

Previous studies evaluated the relationship of CAF specific gene expression with clinical outcome. Several studies showed that a CAF signature is associated with patient prognosis and response to immunotherapy in GC [77,78]. These studies had consistent results indicating that a higher CAF score is associated with poor prognosis, and patients with such a profile were less likely to benefit from immunotherapy. The HEYL gene which is expressed at higher levels in SU group in our study, was included in the prognostic model named CAFS-score for GC [78], thus indicating that tumors with high expression of HEYL are associated with stroma in independent studies.

The major limitation of this study is the sample size utilized for ex vivo validation, as geographical differences in cohorts (especially East Asian vs. others) might affect both SU–SD based, as well as stromal percentage-defined prognostic predictions for gastric cancer. Larger cohorts including patients both within and outside East Asia are needed to conclusively answer these questions. In future experiments IHC-based confirmation of this signature can be performed within specific histopathological tumor subtypes. Specific markers for stromal cells can be utilized to perform double staining to confirm the cell types that express the genes of interest in the tumor microenvironment.

## 5. Conclusions

Our approach based on a transcriptome-wide evaluation of prognostic biomarkers revealed a set of genes that was strongly associated with the tumor stroma, ECM re-organization, and cancer associated fibroblasts; suggests that stromal enrichment is among the most prominent factors related to disease progression and thus prognosis. Therefore, stroma-related gene expression signatures may help predict prognosis and help define treatment strategies following further validation studies.

## Data Availability

Publicly available datasets used in this study can be obtained from NCBI GEO platform (https://www.ncbi.nlm.nih.gov/geo/query/acc.cgi) and NCI GDC data portal (https://portal.gdc.cancer.gov/).

## Supplementary Materials

The following supporting information can be downloaded at: https://www.mdpi.com/article/10.3390/cancers15113035/s1, Figure S1: Hierarchical clustering analysis of TCGA-STAD gastric tumors with prognostic genes; Figure S2: Prognostic analysis of SU–SD groups using disease-free survival as clinical outcome (GSE62254); Figure S3: Hierarchical clustering analysis and Kaplan–Meier graphs of gastric cancer tumors with prognostic genes in independent datasets; Figure S4: Kaplan–Meier graphs comparing patients who received and who did not receive adjuvant chemotherapy; Figure S5: Kaplan–Meier graphs comparing SU–SD groups stratified by treatment status; Figure S6: Kaplan–Meier graphs comparing patients with and without adjuvant chemotherapy stratified by SU–SD groups; Figure S7: Hierarchical clustering analysis with prognostic genes and Kaplan–Meier graphs of gastric cancer tumors in GSE14208 and GSE28541; Figure S8: Enrichment plots of highly enriched gene sets listed in Supplementary Table S6 for (a) GSE62254 and (b) GSE15459; Figure S9: Hierarchical clustering of gastric tumors based on EMT related gene expression; Figure S10: Stromal scores based on ESTIMATE method for SU and SD samples in GSE62254 and GSE15459; Figure S11: Summary graph generated using IPA; Figure S12: Single-cell RNA-seq analysis of 26 gastric tumors (GSE183904); Figure S13: Kaplan–Meier graphs comparing SU–SD groups stratified by TCGA subtypes; Figure S14: Prognostic analysis of SU–SD groups for intestinal and diffuse type diseases, using disease-free survival as clinical outcome (GSE62254); Figure S15: Kaplan–Meier graphs comparing SU–SD groups stratified by ACRG subtypes; Figure S16: Kaplan–Meier graphs comparing SU–SD groups stratified by stage (GSE62254); Figure S17: Hematoxylin and eosin (H&E) stained sections from 12 gastric carcinoma cases with lymphoid stroma; Figure S18: Prognostic relationship of stromal content and TAGLN expression in Turkish gastric cancer patients; Table S1: Patient Characteristics of Turkish cohort (n = 40); Table S2: Primers used in qRT-PCR experiments; Table S3: Antibodies used in immunohistochemistry; Table S4: Cox regression analysis in GSE15459 and GSE62254; Table S5: Multivariate Cox regression analysis—OS (GSE26901); Table S6: Gene set enrichment analysis comparing SU–SD prognostic groups; Table S7: Distribution of the SU and SD samples among EMT related subgroups; Table S8: Distribution of UP-DOWN groups and TCGA subtypes; Table S9: Multivariate analysis of prognostic subgroups and TCGA subtypes-OS; Table S10: Univariate and multivariate Cox regression analysis—OS (GSE62254); Table S11: Univariate and multivariate Cox regression analysis—OS (GSE84437); Table S12: Univariate Cox regression analysis of clinical parameters in discovery and validation cohorts—OS; Table S13: Multivariate Cox regression analysis of prognostic classification and stage in GSE15459-OS; Table S14: Gene–gene linear correlations of IHC-based expression (H-score); Table S15: Correlation of qRT-PCR-based expression levels.
