# Supplementary material for: A Novel Gene List Identifies Tumors with a Stromal-Mesenchymal Phenotype and Worse Prognosis in Gastric Cancer
Source: Cancers (Basel). 2023 Jun 2;15(11):3035. doi: 10.3390/cancers15113035 (PMC10252086; doi:10.3390/cancers15113035)
Supplement: Supplementary file 1 [file Supp Figure S1.pdf › Supp Figure S1.pdf]

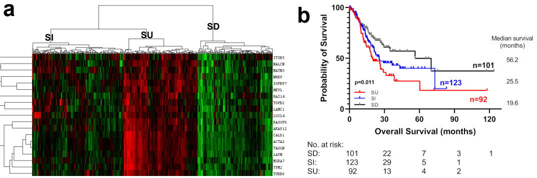

Supplementary Figure S1: Hierarchical clustering analysis of TCGA-STAD gastric tumors with prognostic genes. (a) Normalized RNAseq expression data was used for hierarchical clustering. 19 out of 20 gene names were available. Based on the dendrogram, three major branches labeled as "SI", "SU" and "SD" groups, from left to right, respectively. (b) Kaplan Meier plot for the three groups. Log rank p-value is indicated.
