# Supplementary material for: A Novel Gene List Identifies Tumors with a Stromal-Mesenchymal Phenotype and Worse Prognosis in Gastric Cancer
Source: Cancers (Basel). 2023 Jun 2;15(11):3035. doi: 10.3390/cancers15113035 (PMC10252086; doi:10.3390/cancers15113035)
Supplement: Supplementary file 1 [file Supp Figure S11.pdf › Supp Figure S11.pdf]

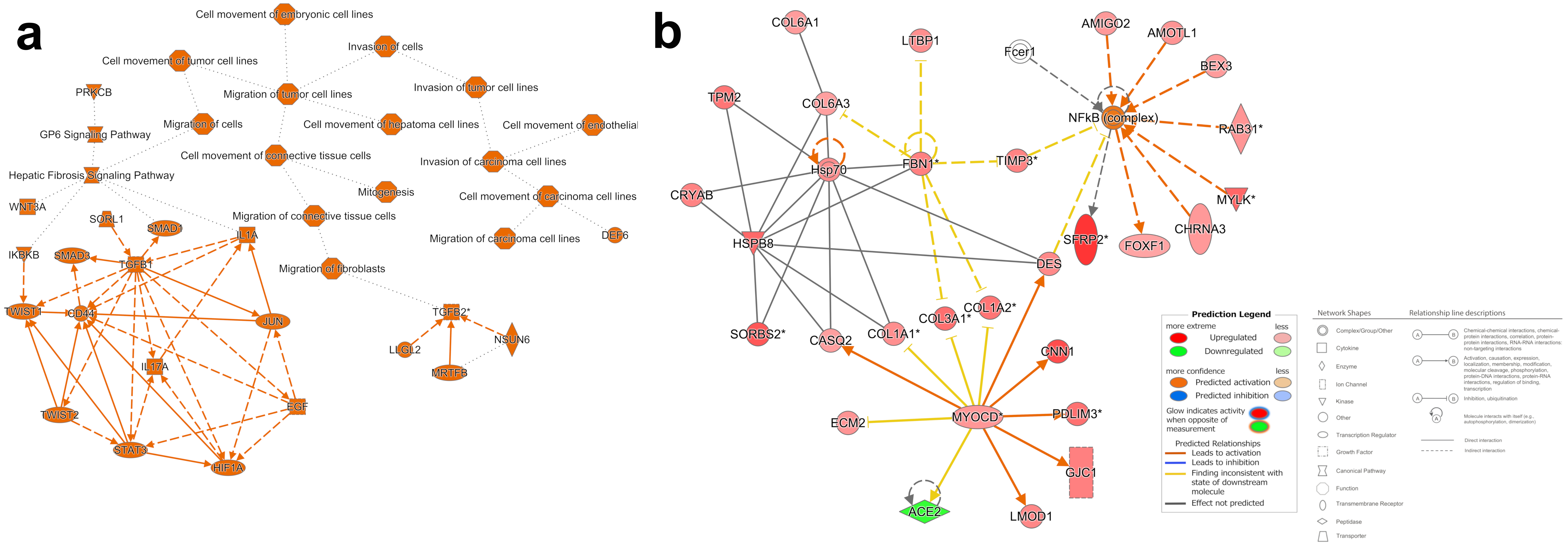

Supplementary Figure S11: Summary graph generated using IPA (QIAGEN Inc., https://digitalinsights.qiagen.com/IPA) (a). Top network with the highest number of focus molecules is given (b).
