# Supplementary material for: A Novel Gene List Identifies Tumors with a Stromal-Mesenchymal Phenotype and Worse Prognosis in Gastric Cancer
Source: Cancers (Basel). 2023 Jun 2;15(11):3035. doi: 10.3390/cancers15113035 (PMC10252086; doi:10.3390/cancers15113035)
Supplement: Supplementary file 1 [file cancers-15-03035-s001.zip › Supp Figure S12.pdf]

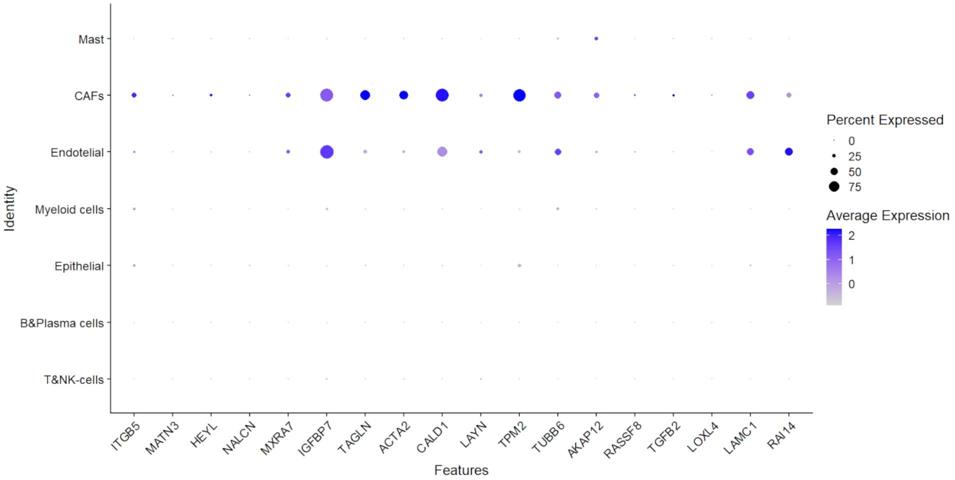

Supplementary Figure S12: Single cell RNA-seq analysis of 26 gastric tumors (GSE183904). 18 genes available in the dataset are shown. Size and color of the dots indicate percentage of cells expressing the gene and average expression, respectively.
