# Supplementary material for: A Novel Gene List Identifies Tumors with a Stromal-Mesenchymal Phenotype and Worse Prognosis in Gastric Cancer
Source: Cancers (Basel). 2023 Jun 2;15(11):3035. doi: 10.3390/cancers15113035 (PMC10252086; doi:10.3390/cancers15113035)
Supplement: Supplementary file 1 [file cancers-15-03035-s001.zip › Supp Figure S13.pdf]

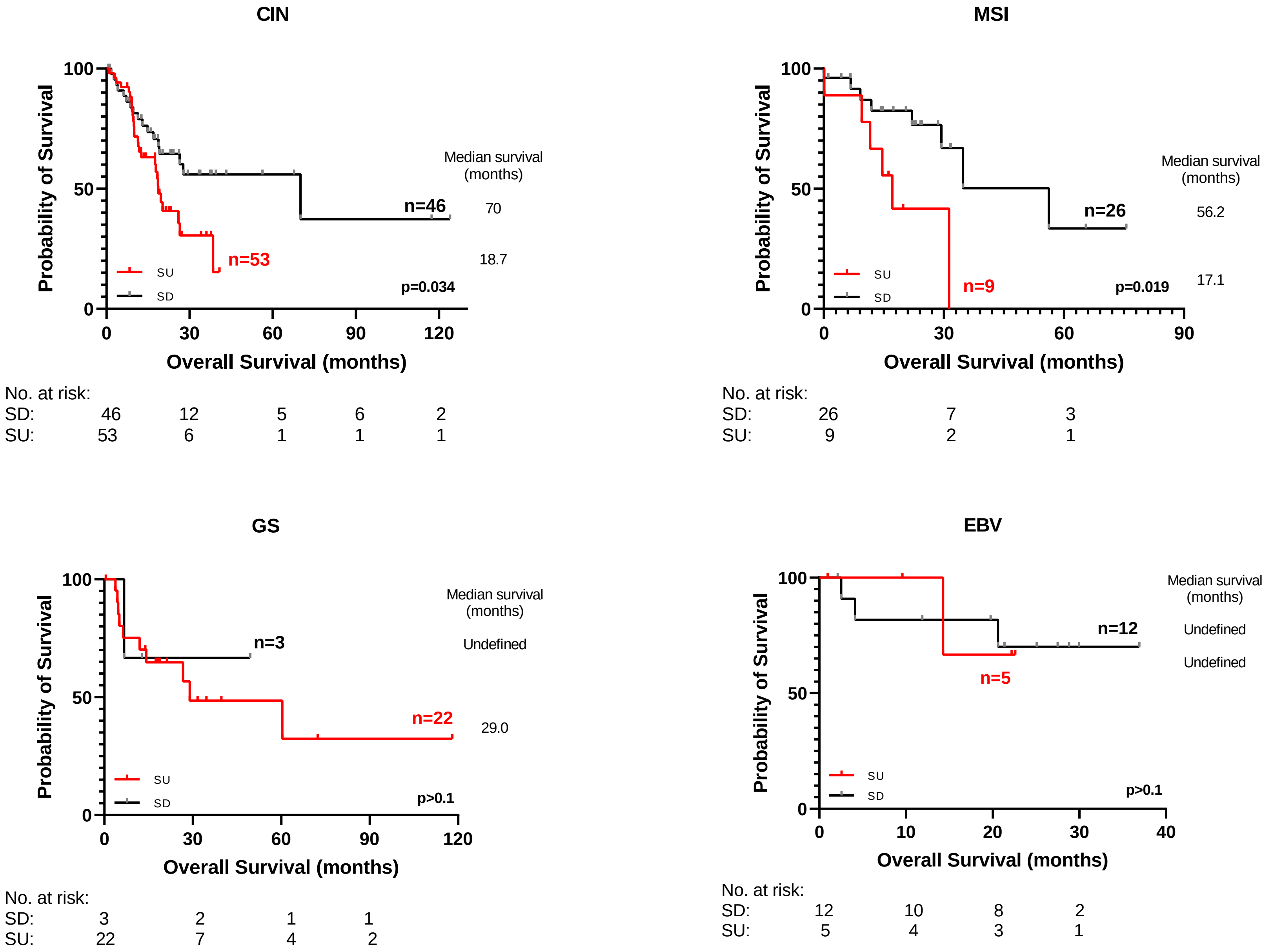

Supplementary Figure S13: Kaplan Meier graphs comparing SU-SD groups stratified by TCGA subtypes. Overall survival was used as clinical outcome. Log-rank p values are indicated. EBV: Tumors positive for Epstein–Barr virus, MSI: microsatellite instable tumors, GS: genomically stable tumors, CIN: tumors with chromosomal instability.
