# Supplementary material for: A Novel Gene List Identifies Tumors with a Stromal-Mesenchymal Phenotype and Worse Prognosis in Gastric Cancer
Source: Cancers (Basel). 2023 Jun 2;15(11):3035. doi: 10.3390/cancers15113035 (PMC10252086; doi:10.3390/cancers15113035)
Supplement: Supplementary file 1 [file cancers-15-03035-s001.zip › Supp Figure S15.pdf]

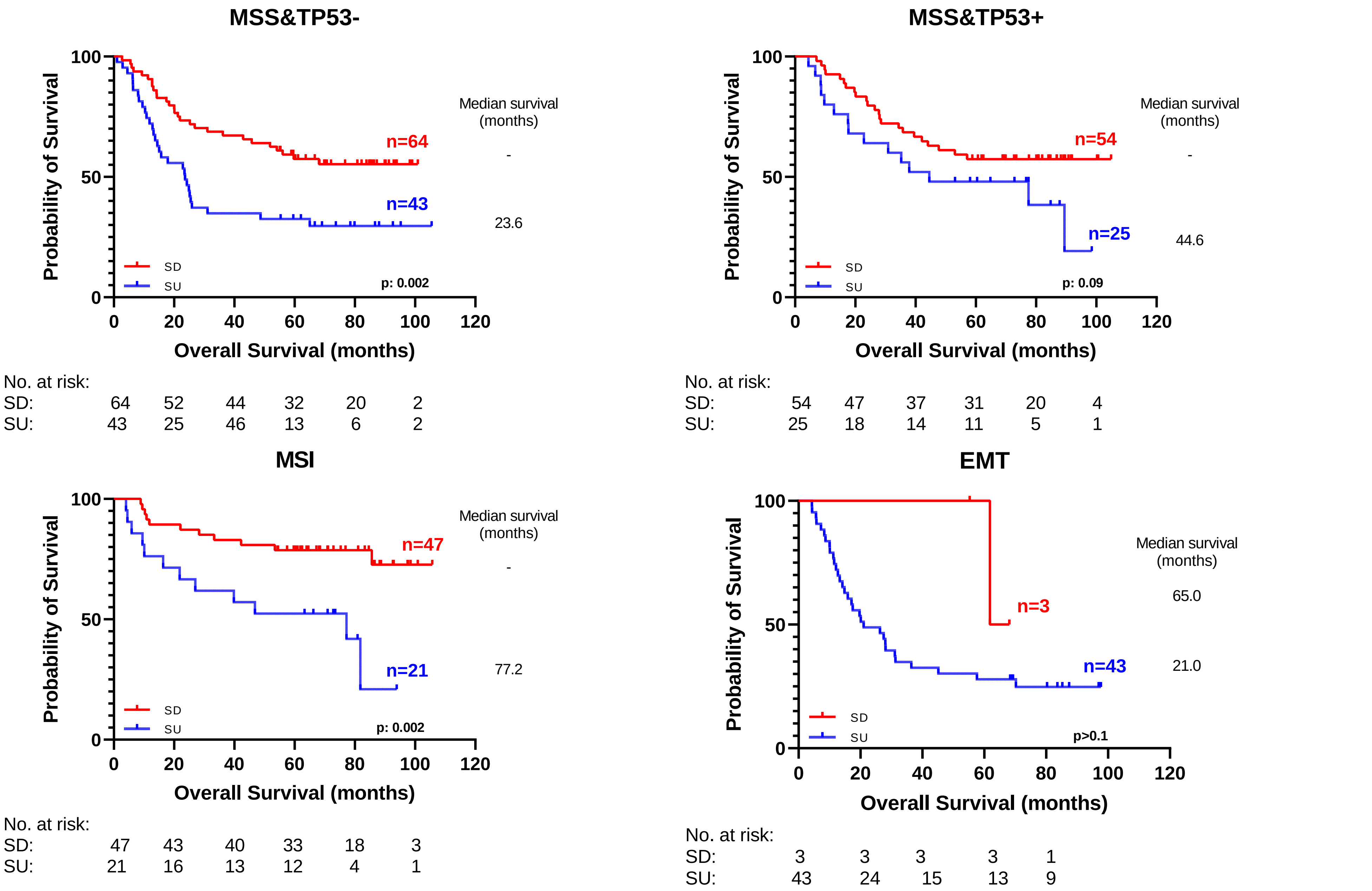


**Supplementary Figure S15:** Kaplan Meier graphs comparing SU-SD groups stratified by ACRG subtypes. Tumor samples in GSE62254 were separated in to four distinct gastric tumor sub-groups previously defined by the Asian Cancer Research Group (ACRG); MSI, MSS/EMT, MSS/TP53+ (MSS with intact TP53 activity), MSS/TP53- (MSS with TP53 functional loss). Prognostic difference between SU and SD groups were evaluated within each group. Overall survival was used as clinical outcome. Log-rank p values are indicated.
