# Supplementary material for: A Novel Gene List Identifies Tumors with a Stromal-Mesenchymal Phenotype and Worse Prognosis in Gastric Cancer
Source: Cancers (Basel). 2023 Jun 2;15(11):3035. doi: 10.3390/cancers15113035 (PMC10252086; doi:10.3390/cancers15113035)
Supplement: Supplementary file 1 [file cancers-15-03035-s001.zip › Supp Figure S16.pdf]

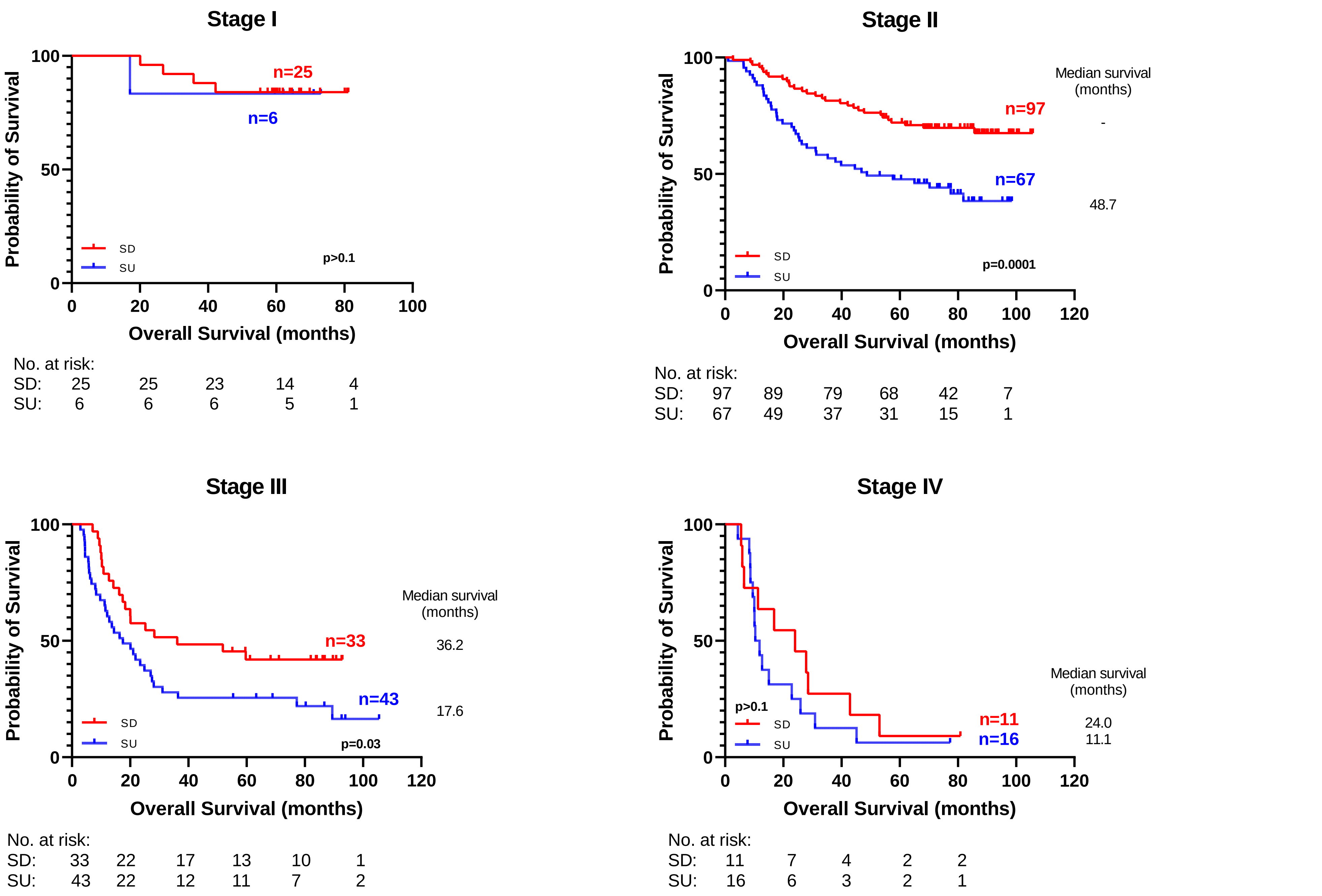

Supplementary Figure S16: Kaplan Meier graphs comparing SU-SD groups stratified by stage (GSE62254). Prognostic difference between SU and SD groups were evaluated within each stage. Overall survival was used as clinical outcome. Log-rank p values are indicated.
