# Supplementary material for: A Novel Gene List Identifies Tumors with a Stromal-Mesenchymal Phenotype and Worse Prognosis in Gastric Cancer
Source: Cancers (Basel). 2023 Jun 2;15(11):3035. doi: 10.3390/cancers15113035 (PMC10252086; doi:10.3390/cancers15113035)
Supplement: Supplementary file 1 [file cancers-15-03035-s001.zip › Supp Figure S2.pdf]

GSE62254 (n=282)

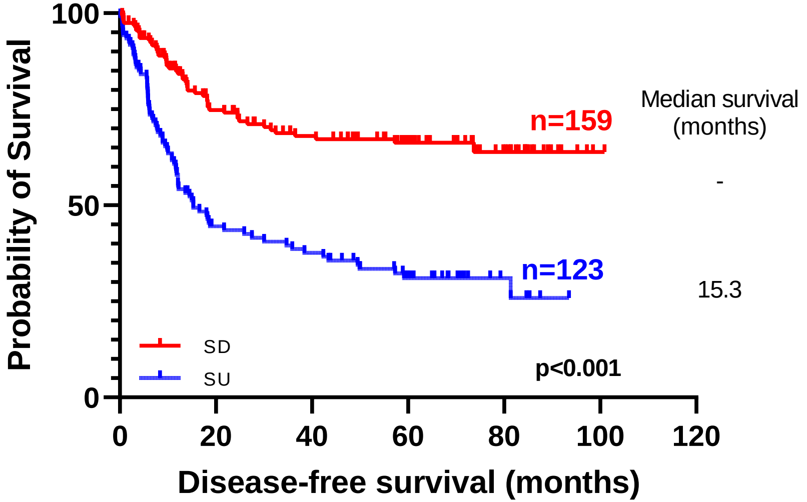

No. at risk:

SD:	159	104	86	51	24	2
SU:	123	46	39	21	7	1

Supplementary Figure S2: Prognostic analysis of SU-SD groups using disease-free survival as clinical outcome (GSE62254). Prognostic difference between SU and SD groups were evaluated. Log-rank p value is indicated.
