# Supplementary material for: A Novel Gene List Identifies Tumors with a Stromal-Mesenchymal Phenotype and Worse Prognosis in Gastric Cancer
Source: Cancers (Basel). 2023 Jun 2;15(11):3035. doi: 10.3390/cancers15113035 (PMC10252086; doi:10.3390/cancers15113035)
Supplement: Supplementary file 1 [file cancers-15-03035-s001.zip › Supp Figure S3.pdf]

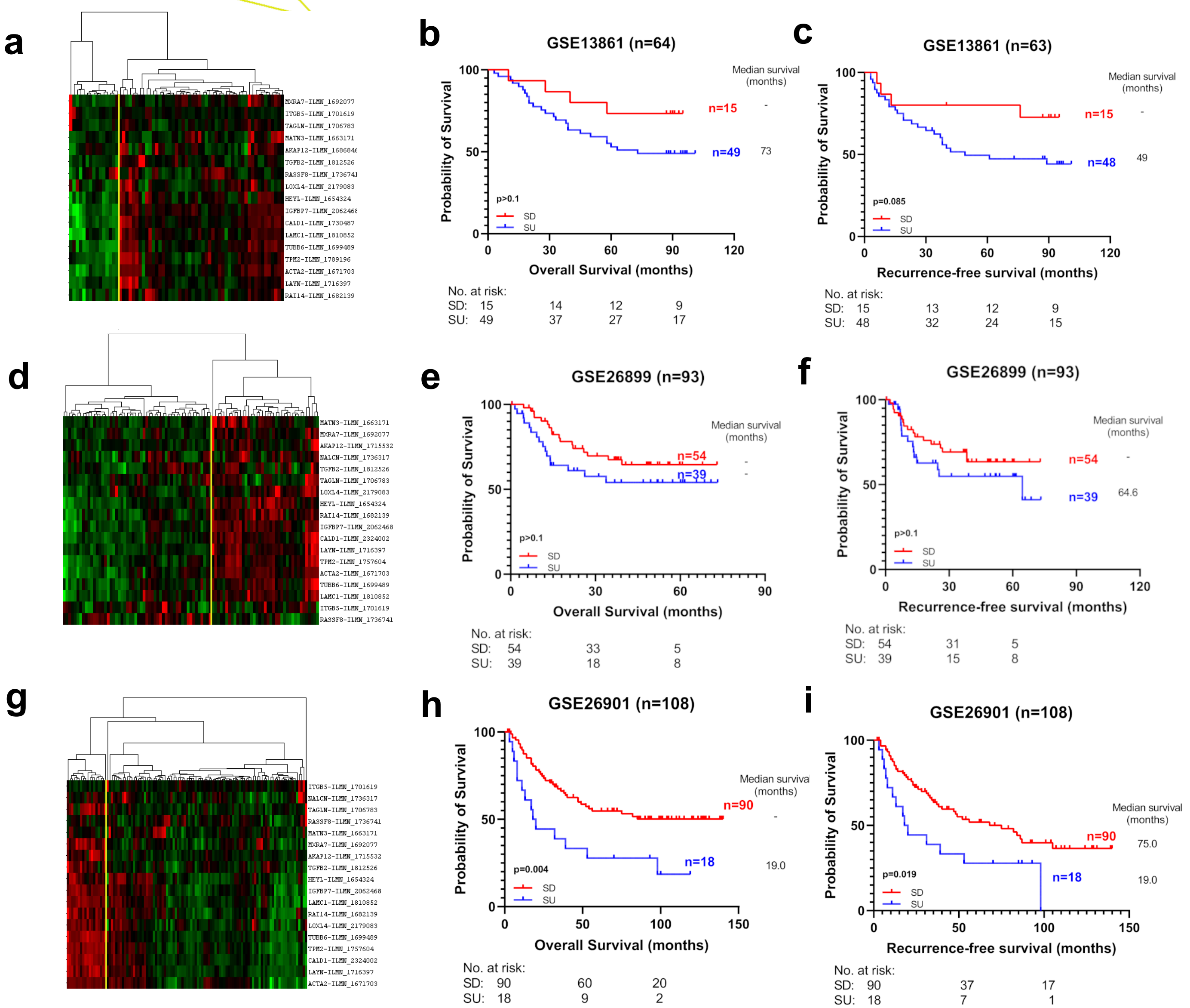

Supplementary Figure S3: Hierarchical clustering analysis and Kaplan-Meier graphs of gastric cancer tumors with prognostic genes in independent datasets. Illumina probesets available for prognostic gene list were used for hierarchical clustering of the three datasets, (a) GSE13861, (d) GSE26899, (g) GSE26901. (b,e,h) Middle panel and (c,f,i) right panel shows Kaplan Meier plots with overall survival and recurrence-free survival, respectively. Major two branches separated by the yellow line were categorized as SU and SD (right: SU, left: SD) for GSE13861 and GSE26899. The tumor on at the right end of the heatmap is excluded and the remaining samples are separated by the yellow line as SU (left) and SD (right) for GSE26901.
