# Supplementary material for: A Novel Gene List Identifies Tumors with a Stromal-Mesenchymal Phenotype and Worse Prognosis in Gastric Cancer
Source: Cancers (Basel). 2023 Jun 2;15(11):3035. doi: 10.3390/cancers15113035 (PMC10252086; doi:10.3390/cancers15113035)
Supplement: Supplementary file 1 [file cancers-15-03035-s001.zip › Supp Figure S4.pdf]

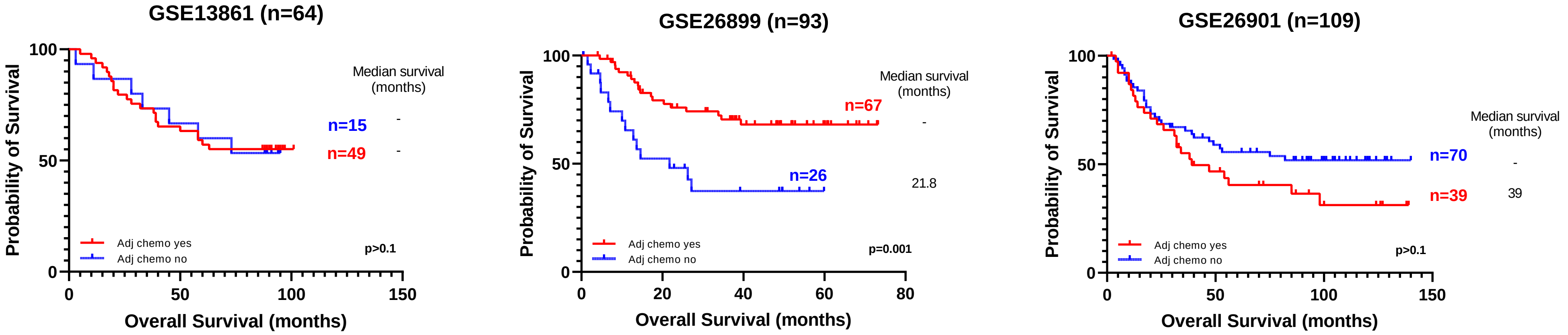

GSE13861

No. at risk:

Adj. chemo yes:	49	32		3
Adj. chemo no:	15	11	1	

GSE26899

No. at risk:

Adj. chemo yes:	67	48	30	12
Adj. chemo no:	26	13	7	1

GSE26901

No. at risk:

Adj. chemo yes:	39	17		6
Adj. chemo no:	70	36	16	

	no	yes
EP	9 (60 %)	39 (79.6%)
MP	6 (40 %)	10 (20.4%)

	no	yes
EP	16 (61.5 %)	50 (74.6%)
MP	10 (38.5 %)	17 (25.4%)

	no	yes
EP	48 (68.6 %)	22 (56.4%)
MP	22 (31.4 %)	17 (43.6%)

Supplementary Figure S4: Kaplan Meier graphs comparing patients who received and who did not receive adjuvant chemotherapy. Distribution of tumors with epithelial (EP) and mesenchymal profiles (MP) obtained from Oh et al [29] is shown across treatment information in GSE13861, GSE26899 and GSE26901.
