# Supplementary material for: A Novel Gene List Identifies Tumors with a Stromal-Mesenchymal Phenotype and Worse Prognosis in Gastric Cancer
Source: Cancers (Basel). 2023 Jun 2;15(11):3035. doi: 10.3390/cancers15113035 (PMC10252086; doi:10.3390/cancers15113035)
Supplement: Supplementary file 1 [file cancers-15-03035-s001.zip › Supp Figure S5.pdf]

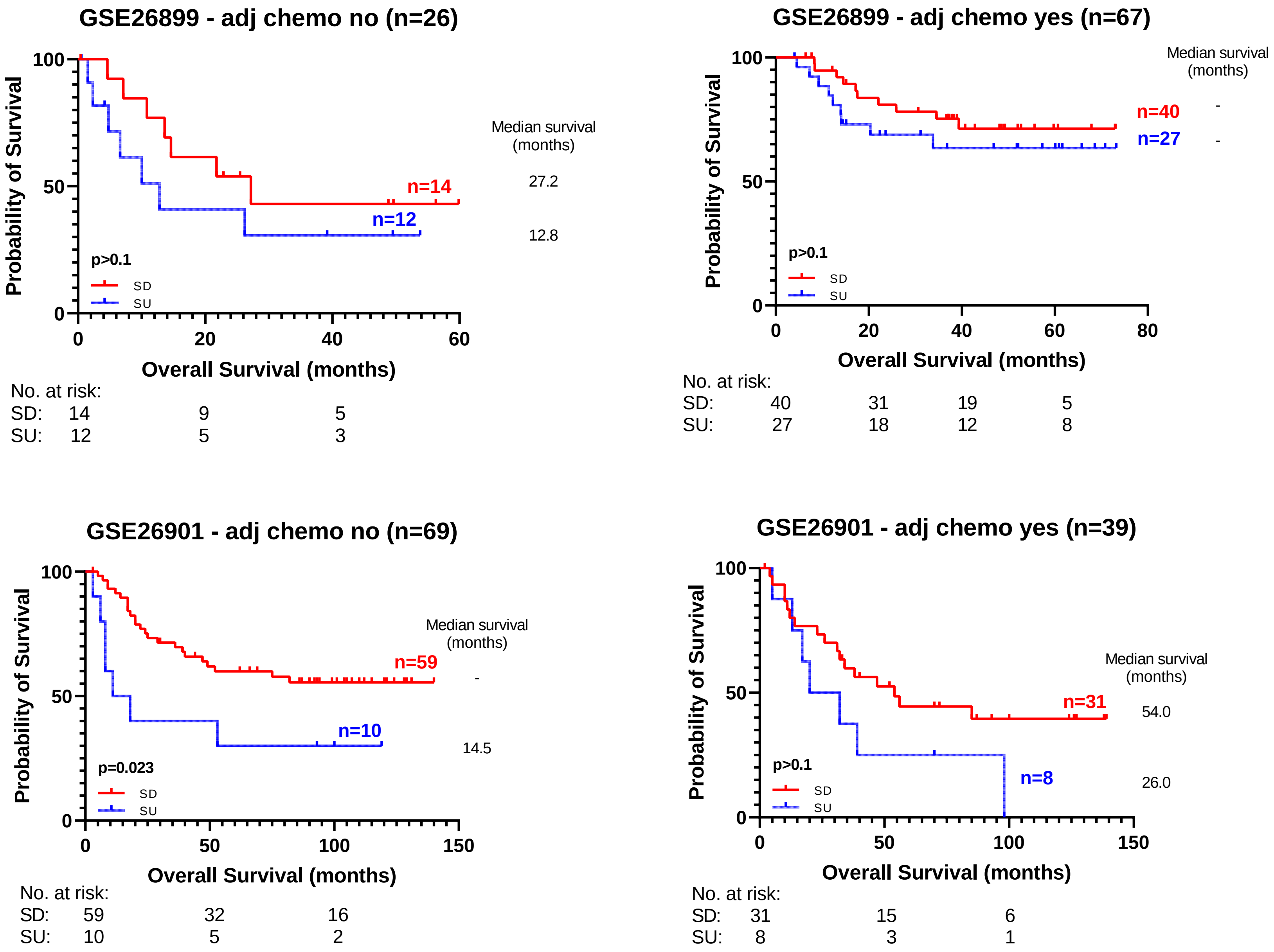

Supplementary Figure S5: Kaplan Meier graphs comparing SU-SD groups stratified by treatment status. Patients who received adjuvant chemotherapy (right panel) and patients who did not receive adjuvant chemotherapy were included in the prognostic analysis. GSE26899 and GSE26901 are shown in the upper and lower panels, respectively. Log-rank p values are indicated.
