# Supplementary material for: A Novel Gene List Identifies Tumors with a Stromal-Mesenchymal Phenotype and Worse Prognosis in Gastric Cancer
Source: Cancers (Basel). 2023 Jun 2;15(11):3035. doi: 10.3390/cancers15113035 (PMC10252086; doi:10.3390/cancers15113035)
Supplement: Supplementary file 1 [file cancers-15-03035-s001.zip › Supp Figure S6.pdf]

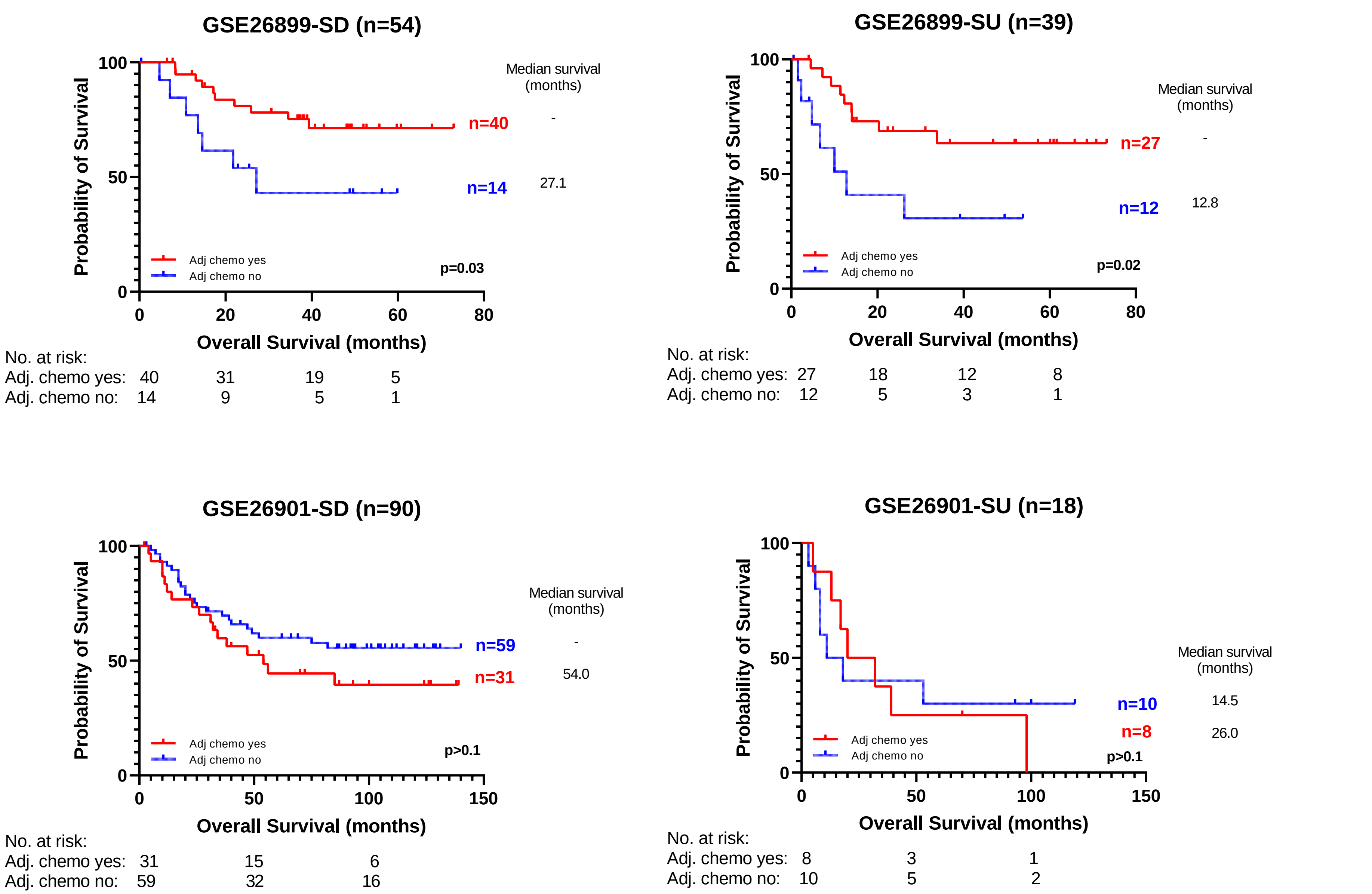

Supplementary Figure S6: Kaplan Meier graphs comparing patients with and without adjuvant chemotherapy stratified by SU-SD groups. Prognostic difference between patients who received and who did not receive adjuvant chemotherapy were evaluated within SU and SD groups separately. GSE26899 and GSE26901 are shown in the upper and lower panels, respectively. Log-rank p values are indicated.
