# Supplementary material for: A Novel Gene List Identifies Tumors with a Stromal-Mesenchymal Phenotype and Worse Prognosis in Gastric Cancer
Source: Cancers (Basel). 2023 Jun 2;15(11):3035. doi: 10.3390/cancers15113035 (PMC10252086; doi:10.3390/cancers15113035)
Supplement: Supplementary file 1 [file cancers-15-03035-s001.zip › Supp Figure S7.pdf]

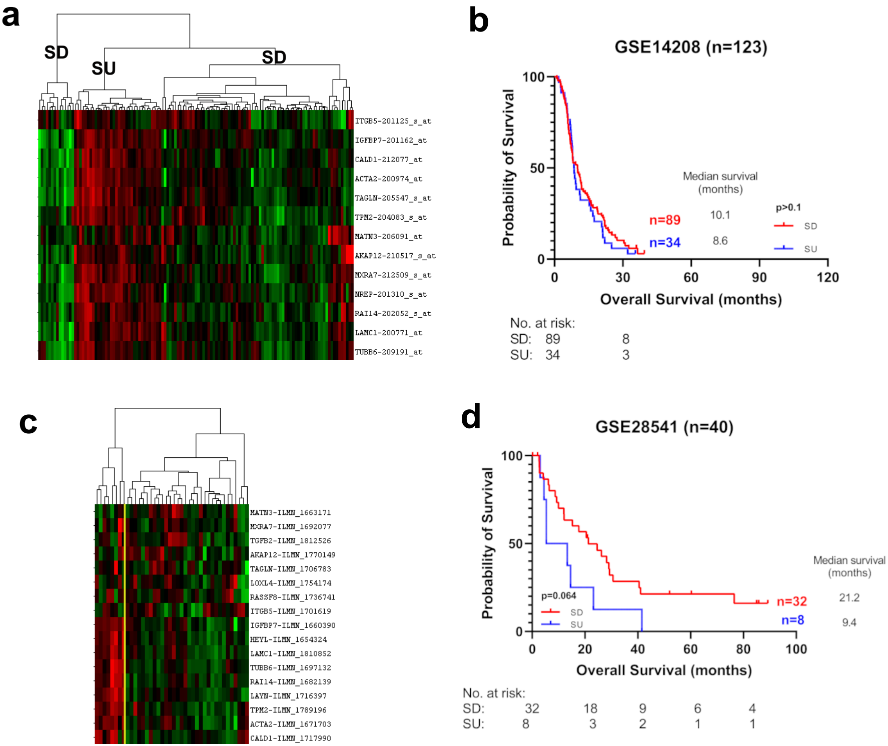

Supplementary Figure S7: Hierarchical clustering analysis with prognostic genes and Kaplan-Meier graphs of gastric cancer tumors in GSE14208 and GSE28541. (a) 13 out of 20 probesets available in GSE14208 were used and "SU" and "SD" groups were labeled based on expression patterns. (c) 17 illumina probesets matching with prognostic genes in GSE28541 were used for hierarchical clustering analysis. Major two branches separated by the yellow line were categorized as SU and SD (left: SU, right: SD). Kaplan Meier graphs are shown for (b) GSE14208 (d) GSE28541.
