# Supplementary material for: A Novel Gene List Identifies Tumors with a Stromal-Mesenchymal Phenotype and Worse Prognosis in Gastric Cancer
Source: Cancers (Basel). 2023 Jun 2;15(11):3035. doi: 10.3390/cancers15113035 (PMC10252086; doi:10.3390/cancers15113035)
Supplement: Supplementary file 1 [file cancers-15-03035-s001.zip › Supp Figure S8.pdf]

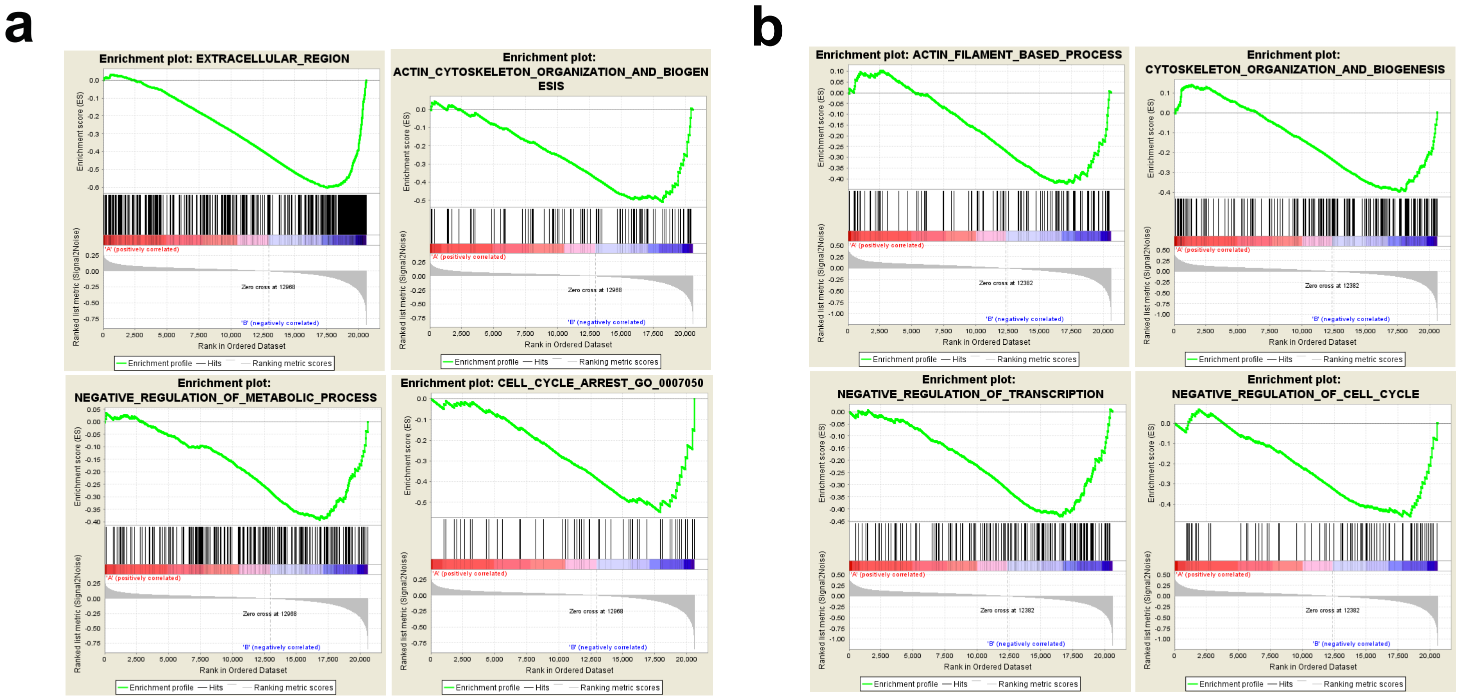

Supplementary Figure S8: Enrichment plots of highly enriched gene sets listed in Supplemantary Table S6 for (a) GSE62254 and (b) GSE15459. Genesets related to the extracellular matrix, cytoskeleton and a reduction of metabolic processes and cell growth were noted. The groups in the enrichment plot are A: SD, B: SU.
