# Supplementary material for: A Novel Gene List Identifies Tumors with a Stromal-Mesenchymal Phenotype and Worse Prognosis in Gastric Cancer
Source: Cancers (Basel). 2023 Jun 2;15(11):3035. doi: 10.3390/cancers15113035 (PMC10252086; doi:10.3390/cancers15113035)
Supplement: Supplementary file 1 [file cancers-15-03035-s001.zip › Supp Figure S9.pdf]

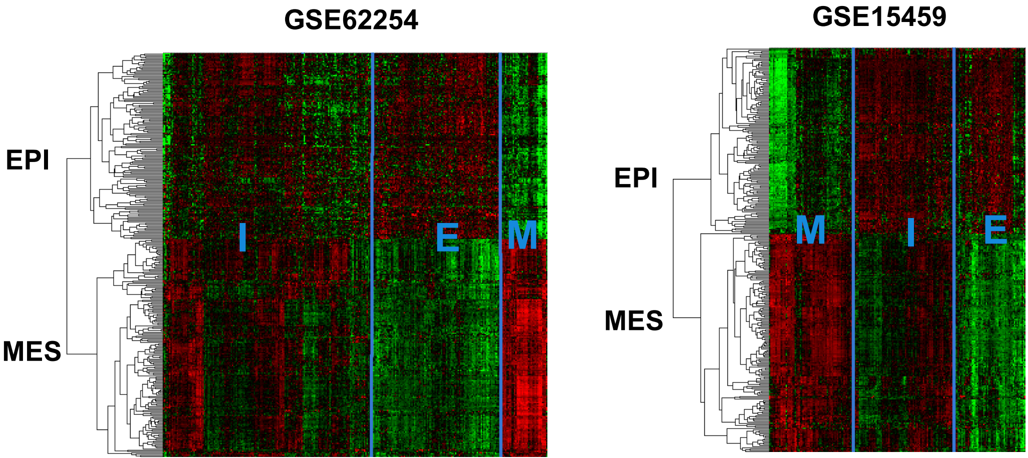

Supplementary Figure S9: Hierarchical clustering of gastric tumors based on EMT related gene expression. Rows: Probesets, Columns: Tumors. EPI: Epithelial markers, MES: Mesenchymal markers. Tumors with high epithelial marker and low mesenchymal marker expression were named as E (Epithelial), tumors with the opposite pattern were named as M (Mesenchymal), the rest of the samples were named as I (Intermediate).
