# Supplementary material for: A Novel Gene List Identifies Tumors with a Stromal-Mesenchymal Phenotype and Worse Prognosis in Gastric Cancer
Source: Cancers (Basel). 2023 Jun 2;15(11):3035. doi: 10.3390/cancers15113035 (PMC10252086; doi:10.3390/cancers15113035)
Supplement: Supplementary file 1 [file cancers-15-03035-s001.zip › Supp. Figure S17.pdf]

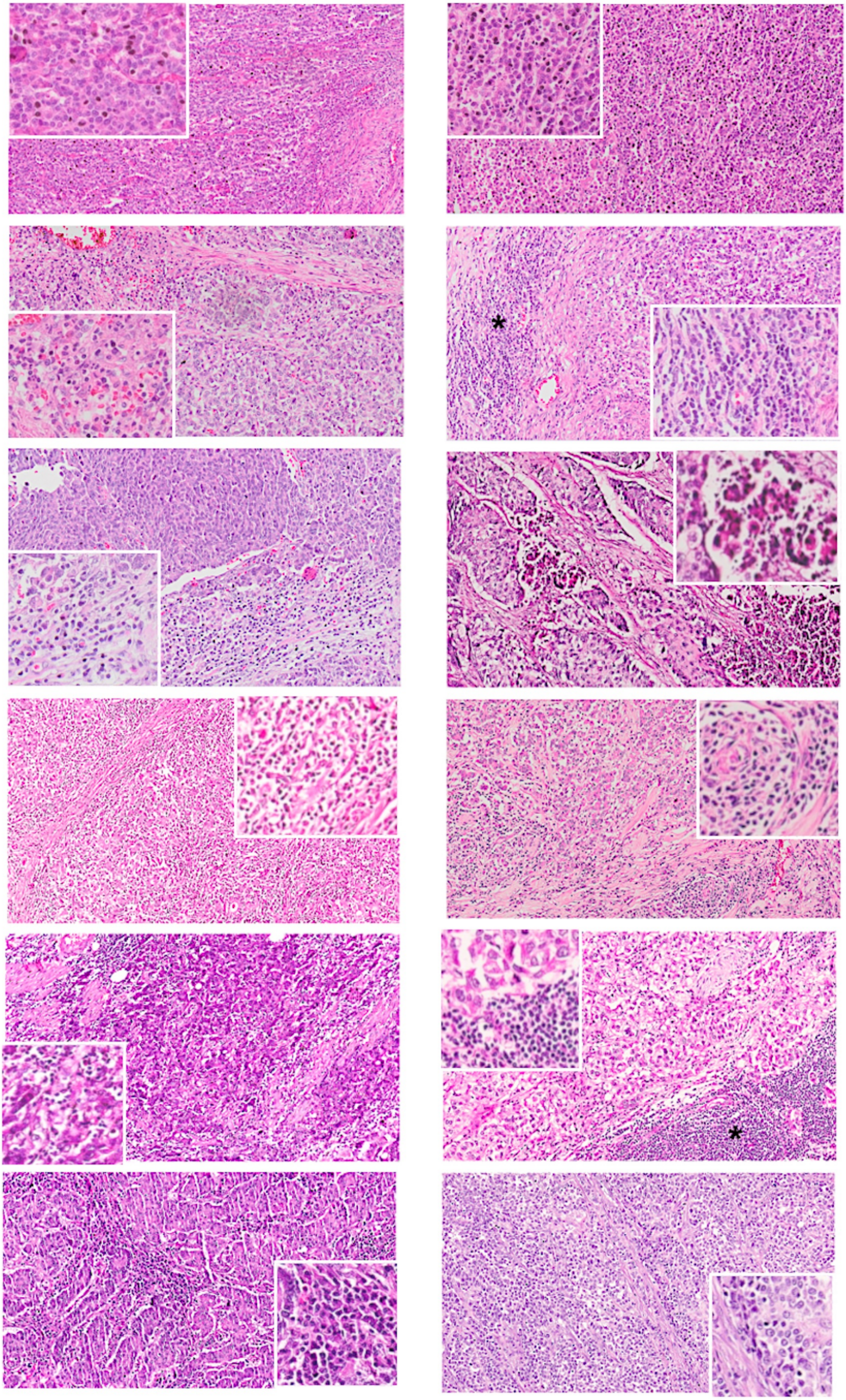

Supplementary Figure S17: Hematoxylin and eosin (H&E) stained sections from 12 gastric carcinoma cases with lymphoid stroma. All 12 tumors show medullary pattern with sheets and/or trabeculae of syncytial polygonal neoplastic cells embedded within a lymphocyte rich stroma (representative lymphoid rich regions are indicated with asterisks). Neoplasms lack prominent glandular differentiation and frequently contain intraepithelial lymphocytes.
