# Supplementary material for: A Novel Gene List Identifies Tumors with a Stromal-Mesenchymal Phenotype and Worse Prognosis in Gastric Cancer
Source: Cancers (Basel). 2023 Jun 2;15(11):3035. doi: 10.3390/cancers15113035 (PMC10252086; doi:10.3390/cancers15113035)
Supplement: Supplementary file 1 [file cancers-15-03035-s001.zip › Supp. Figure S18.pdf]

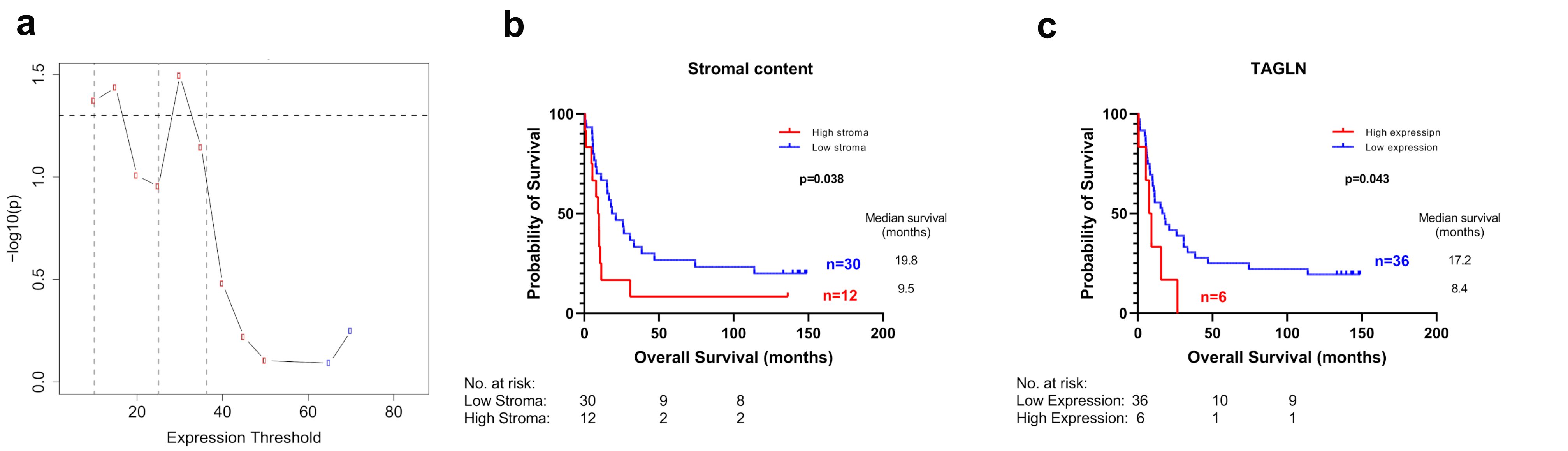

Supplementary Figure S18: Prognostic relationship of stromal content and TAGLN expression in Turkish gastric cancer patients. (a) Relationship of stromal content (percent of stroma in the examined area) and overall survival was evaluated based on log-rank p values plotted on the y axis (-log10(p)), comparing the tumors with “high” and “low” stromal contents generated based on all possible cut-offs shown on the x axis. Red and blue colors indicate association of high expression with poor and good prognosis, respectively. Vertical dashed lines represent 25 percentile, median and 75th percentile values. Horizontal dashed line indicates 0.05 p value. (b) Kaplan Meier plot comparing tumors with high (>30) and low (≤30) stromal contents. (c) Patients with high (H score > 10) and low (H score ≤ 10) IHC based TAGLN expression were compared using Kaplan Meier plot. Log rank p-values are indicated.
