# Supplementary material for: A Novel Gene List Identifies Tumors with a Stromal-Mesenchymal Phenotype and Worse Prognosis in Gastric Cancer
Source: Cancers (Basel). 2023 Jun 2;15(11):3035. doi: 10.3390/cancers15113035 (PMC10252086; doi:10.3390/cancers15113035)
Supplement: Supplementary file 1 [file cancers-15-03035-s001.zip › Supplementary Tables.pdf]

cancers

A Novel Gene List Identifies Tumors with a Stromal-Mesenchymal Phenotype and Worse Prognosis in Gastric Cancer

Secil Demirkol Canli, Meral Uner, Baris Kucukkaraduman, Diren Arda Karaoglu, Aynur Isik, Nesrin Turhan, Aytekin Akyol, Ismail Gomceli and Ali Osmay Gure

Table S1. Patient Characteristics of Turkish cohort (n = 40) *.

Age	Nr.**	Percentage
above 60	26	65.0 %
below 60	14	35.0 %
Gender		
Male	26	65.0 %
Female	14	35.0 %
TNM Stage		
1B	3	7.5 %
2A	11	27.5 %
2B	12	30.0 %
3A	3	7.5 %
3B	4	10.0 %
4	4	10.0 %
NA	3	7.5 %
Differentiation		
low	20	50.0 %
moderate	10	25.0 %
high	5	12.5 %
NA	5	12.5 %
Perineural invasion		
yes	25	62.5 %
no	14	35.0 %
NA	1	2.5 %
Lymphovascular invasion		
yes	32	80.0 %
no	7	17.5 %
NA	1	2.5 %
MMR status		
pMMR	25	62.5%
eqMMR	7	17.5 %
dMMR	7	17.5 %
NA	1	2.5 %

* Two patients who did not have paraffin blocks are not included.
** Number of patients

Table S2. Primers used in qRT-PCR experiments.

**ACTA2 F**	**ACTGCCTTGGTGTGTGACAA**
ACTA2 R	CACCATCACCCCCTGATGTC
CALD1 F	TCGACCCATCAAGCAGCAA
CALD1 R	TTTGCGCTTTTTGTTCCCTCAA
HEYL F	CATCGACGTGGGCCAAGAG
HEYL R	GCCGTTTCTCTATGATCCCTCTG
TAGLN F	CTATGGCATGAGCCGCGAAG
TAGLN R	ACCAGCTTGCTCAGAATCACG
TPM2 F	GAAGCCGACAAGAAGCAAGC
TPM2 R	ATCTGCCTCAGCATCAGTGG
CDH1 F	TCCAGGAACCTCTGTGATGGA
CDH1 R	CGTAGGGAAACTCTCTCGGTC
VIM F	CGGGAGAAATTGCAGGAGGA
VIM R	AAGGTCAAGACGTGCCAGAG
B2M F	TAAGCAGCATCATGGAGGTTTG
B2M R	AGCAAGCAAGCAGAATTTGGA
GAPDH F	TCGGAGTCAACGGATTTGGT
GAPDH R	TTCCCGTTCTCAGCCTTGAC

Table S3. Antibodies used in immunohistochemistry.

**Antibody**	**Antigen/ Epitope Retrieval Reagent**	**Dilution**	**Clone**	**Trademark /Vendor**
HEYL	EDTA	1:250	HPA076960*	SIGMA ALDRICH
CALD1	Citrate	1:2000	HPA017330*	SIGMA ALDRICH
TAGLN	Citrate	1:100	HPA019467*	SIGMA ALDRICH
ACTA2 (*α-SMA*)	Citrate	1:1000	1a4 (asm-1)	NEOMARKERS
TMP2	EDTA	1:25	HPA053624*	SIGMA ALDRICH
MLH1	EDTA	1:50	ES05	Novocastra
MSH2	EDTA	1:100	79H11	Novocastra
MSH6	EDTA	1:100	PU29	Novocastra
PMS2	EDTA	1:100	M0R4G	Novocastra

*Antibody is selected based on the staining patterns and matching transcripts information https://www.proteinatlas.org.

Table S4. Cox regression analysis in GSE15459 and GSE62254.

		GSE15459			GSE62254			
**Probeset**	**Gene**	**HR**	**Cox p**	**RANK coxp**	**HR**	**Cox p**	**RANK coxp**	**RANK SUM coxp**
226828_s_at	HEYL	1.702609	8.58E-06	52	1.697446	3.1E-10	25	77
201162_at	IGFBP7	1.741568	0.000119	198	1.892259	4.47E-08	207	405
229669_at	LOC339260	1.449439	6.35E-06	46	1.346171	2.06E-07	362	408
201125_s_at	ITGB5	3.080442	3.11E-07	10	2.37884	4.02E-07	453	463
206091_at	MATN3	1.387757	1.85E-05	71	1.420368	2.82E-07	397	468
228608_at	NALCN	1.732879	9.04E-08	4	1.544278	6.89E-07	515	519
212509_s_at	MXRA7	1.381559	0.001038	607	1.691716	1.23E-10	13	620
210517_s_at	AKAP12	1.303829	0.000299	312	1.330301	1.41E-07	321	633
228121_at	TGFB2	1.443572	0.000199	252	1.489531	2.52E-07	388	640
227145_at	LOXL4	1.665041	0.000787	522	1.823658	1.06E-08	121	643
205547_s_at	TAGLN	1.277949	0.000935	567	1.331379	4.83E-09	80	647
201310_s_at	NREP	1.482584	0.000613	454	1.589381	5.12E-08	224	678
204083_s_at	TPM2	1.220071	0.001071	623	1.378113	5.19E-09	86	709
209191_at	TUBB6	1.335698	0.001102	634	1.619157	5.19E-09	87	721
200771_at	LAMC1	1.439938	0.001314	705	2.121746	5.05E-10	30	735
200974_at	ACTA2	1.36725	0.001223	676	1.52945	4.38E-09	76	752
212077_at	CALD1	1.462355	0.000262	289	1.644855	4.76E-07	470	759
202052_s_at	RAI14	1.67207	2.01E-05	75	1.651876	1.68E-06	694	769
225946_at	RASSF8	1.24281	0.001026	602	1.426558	3.34E-08	185	787
228080_at	LAYN	1.295407	0.001005	592	1.418032	4.32E-08	202	794

Table S5. Multivariate cox regression analysis – OS (GSE26901).

					95.0% CI for Exp(B)	
		**Number**	**p value**	**HR**	**Lower**	**Upper**
Step 1	**SU-SD**		**0.01**	0.444	0.24	0.82
	SU (ref)	18				
	SD	90				
	**Adjuvant chemotherapy**		0.228	0.718	0.418	1.231
	Yes (ref)	39				
	No	69				
Step 2	**SU-SD**		**0.006**	0.423	0.23	0.776

Table S6. Gene set enrichment analysis comparing SU-SD prognostic groups.

GSE62254*		
**NAME**	**FDR q-val**	**NES**
EXTRACELLULAR_REGION	0	-0.6
ACTIN_CYTOSKELETON_ORGANIZATION_AND_BIOGENESIS	0.004	-0.51
ACTIN_BINDING	0.012	-0.53
CYTOSKELETON	0	-0.39
EXTRACELLULAR_REGION_PART	0	-0.63
ACTIN_CYTOSKELETON	0.006	-0.5
PROTEINACEOUS_EXTRACELLULAR_MATRIX	0	-0.78
EXTRACELLULAR_MATRIX	0	-0.77
EXTRACELLULAR_MATRIX_PART	0	-0.8
WOUND_HEALING	0.017	-0.6
CELL_MIGRATION	0	-0.63
NEGATIVE_REGULATION_OF_METABOLIC_PROCESS	0	-0.39
CELL_CYCLE_ARREST_GO_0007050	0.004	-0.55
NEGATIVE_REGULATION_OF_CELL_CYCLE	0.002	-0.49

GSE15459*		
**NAME**	**FDR q-val**	**NES**
ACTIN_FILAMENT_BASED_PROCESS	0.03	-0.42
CYTOSKELETON_ORGANIZATION_AND_BIOGENESIS	0.006	-0.4
ACTIN_CYTOSKELETON_ORGANIZATION_AND_BIOGENESIS	0.034	-0.44
ACTIN_BINDING	0.05	-0.47
EXTRACELLULAR_MATRIX	0.016	-0.69
PROTEINACEOUS_EXTRACELLULAR_MATRIX	0.018	-0.7
CYTOSKELETAL_PROTEIN_BINDING	0.004	-0.47
EXTRACELLULAR_MATRIX_PART	0.01	-0.72
WOUND_HEALING	0.04	-0.53
NEGATIVE_REGULATION_OF_TRANSCRIPTION	0.002	-0.43
NEGATIVE_REGULATION_OF_CELL_CYCLE	0.01	-0.46
NEGATIVE_REGULATION_OF_CELLULAR_METABOLIC_PROCESS	0.014	-0.38
NEGATIVE_REGULATION_OF_METABOLIC_PROCESS	0.018	-0.38
CELL_CYCLE_ARREST_GO_0007050	0.004	-0.54

* Enriched gene sets with a consistent pattern of biology were listed in GSE62254 and GSE15459 datasets. FDR q values below 0.25 were considered significant.

Table S7. Distribution of the SU and SD samples among EMT related sub-groups.

GSE62254*			GSE15459*		
	**SU**	**SD**		**SU**	**SD**
**E**	12	92	**E**	0	39
**I**	86	74	**I**	35	55
**M**	34	2	**M**	58	5

* Chi square $p < 0.00001$ for both datasets.

Table S8. Distribution of SU-SD groups and TCGA subtypes.

	TCGA subtype				
	**STAD_CIN**	**STAD_EBV**	**STAD_GS**	**STAD_MSI**	**TOTAL**
**SU**	63 (30.4%) (58.3%)*	5 (18.5%) (4.6%)*	27 (60.0%) (25.0%)*	13 (21.3%) (12.0%)*	108 (100%)
**SI**	90 (43.5%) (68.7%)*	9 (33.3%) (6.9%)*	14 (31.1%) (10.7%)*	18 (29.5%) (13.7%)*	131 (100%)
**SD**	54 (26.1%) (53.4%)*	13 (48.1%) (12.9%)*	4 (8.9%) (4.0%)*	30 (49.2%) (29.7%)*	101 (100%)
TOTAL	207 (100%)	27 (100%)	45 (100%)	61 (100%)	

Number of samples, percentage among TCGA subtypes, and percentage among SU-SI-SD groups (*) are shown.
Chi square $p < 0.001$

Table S9. Multivariate analysis of prognostic subgroups and TCGA subtypes -OS.

	**No. of patients**	**Percentage**	**HR**	**95% CI Lower**	**95% CI Upper**	**p-value**
**Prognostic groups**						
SD (ref.)	90	30.50%				**0.027**
SI vs SD	115	38.98%	1.368	0.852	2.196	0.195
SU vs SD	90	30.50%	1.965	1.194	3.236	**0.008**
**TCGA subtypes**						
MSI (ref.)	48	16.60%				0.579
CIN vs MSI	177	73.40%	1.332	0.79	2.246	0.282
EBV vs MSI	26	32.30%	1.242	0.555	2.777	0.598
GS vs MSI	38	29.70%	0.987	0.485	2.01	0.971

Table S10. Univariate and multivariate cox regression analysis – OS (GSE62254).

GSE62254			Univariate				Multivariate			
	**No. of patients**	**Percentage**	**HR**	**95% CI Lower**	**95% CI Upper**	**p-value**	**HR**	**95% CI Lower**	**95% CI Upper**	**p-value**
**Gender**										
Female	101	33.67%	1.105	.790	1.545	.559				
Male	199	66.33%								
**Age**										
>60	183	61.00%	.792	.568	1.104	.168				
<60	117	39.00%								
**Tumor subtype**										
intestinal (ref.)	150	50.00%				**.004**				**.034**
diffuse vs intestinal	142	47.33%	1.746	1.258	2.424	**.001**	.802	.429	1.501	.491
mixed vs intestinal	8	2.67%	1.689	.679	4.205	.260	6.642	1.436	30.723	**.015**
**Location**										
antrum (ref.)	155	51.67%				**.013**				.757
body	107	35.67%	1.091	.765	1.555	.631	1.253	.630	2.492	.520
cardia	32	10.67%	1.661	1.021	2.702	**.041**	1.334	.462	3.852	.594
whole	6	2.00%	3.273	1.416	7.566	**.006**	.659	.161	2.706	.563
**H.pylori infection**										
no	72	24.00%	1.161	.686	1.966	.579	1.622	.892	2.947	.113
yes	55	18.33%								
notchecked*	173	57.67%								
**AJCC stage**										
1	31	10.33%	2.283	1.875	2.780	**<0.001**	3.025	1.973	4.638	**<0.001**
2	164	54.67%								
3	76	25.33%								
4	27	9.00%								
**MLH1 IHC**										
Negative	64	21.33%				**0.027**				.122
positive	233	77.67%					7.879	.826	75.178	.073
partial loss*	2	0.67%					2.109	.779	5.708	.142
positive; MSH2 mutation (+)*	1	0.33%								
**Mol. Subtype**										
EMT (ref.)	46	15.33%				**.000**				.341
MSI	68	22.67%	.332	.195	.566	**.000**	2.586	.789	8.475	.117
MSS/TP53-	107	35.67%	.640	.417	.982	**.041**	.979	.420	2.279	.960
MSS/TP53+	79	26.33%	.502	.315	.801	**.004**	1.117	.440	2.832	.816
**EBV ISH**										
positive	18	6.00%				.619				
negative	257	85.67%								
NA*	25	8.33%								
**SU-SD Sub-groups**										
SD	168	56%	2.486	1.798	3.438	**<0.001**	2.115	1.102	4.060	**.024**
SU	132	44%								

* Not included in the analysis.

Table S11. Univariate and multivariate cox regression analysis – OS (GSE84437).

**GSE84437**			**Univariate**				**Multivariate**			
	**No. of patients**	**Percentage**	**HR**	**95% CI Lower**	**95% CI Upper**	**p-value**	**HR**	**95% CI Lower**	**95% CI Upper**	**p-value**
**Gender**										
Female	137	31.79%	0.808	0.597	1.094	0.168				
Male (ref)	294	68.21%								
**Age**										
>60	237	54.99%	1.324	1.148	1.527	**<0.001**	1.848	1.388	2.46	**<0.001**
<60 (ref)	194	45.01%								
**pT stage**										
T1, T2 (ref)	49	11.37%	3.708	1.901	7.235	**<0.001**	3.054	1.553	6.003	**0.001**
T3, T4	382	88.63%								
**pN stage**										
N1, N2 (ref)	399	92.58%	2.317	1.37	3.334	**0.001**	2.035	1.298	3.191	**0.002**
N3, N4	32	7.42%								
**SU-SD Sub-groups**										
SD	220	51.04%	1.809	1.37	2.388	**0.001**	1.794	1.352	2.382	**<0.001**
SU	211	48.96%								

Table S12. Univariate Cox regression analysis of clinical parameters in discovery and validation cohorts – OS.

	**DISCOVERY**		**VALIDATION**	
	**GSE15459 (n=192)**		**GSE29272 (n=126)**	
	**HR**	**p-value**	**HR**	**p-value**
**Stage***	2.789	**<0.001**	1.155	0.451
**Age (< or > 60)**	0.983	0.936	1.407	0.098
**Gender (Female or male)**	0.713	0.127	1.122	0.642
**Tumor subtype****		0.578		0.134

* Stage: treated as a continuous variable (1, 2, 3, 4).
** Tumor subtype: treated as a categorical variable (intestinal, mixed, diffuse)

Table S13. Multivariate cox regression analysis of prognostic classification and stage in GSE15459-OS.

**GSE15459**	**p-value**	**HR**	**95% CI for HR**	
			**Lower**	**Upper**
**SD vs SU**	**0.002**	0.493	0.316	0.770
**Stage***	<0.001	2.608	1.997	3.406

* Stage: treated as a continuous variable (1, 2, 3, 4).

Table S14. Gene-gene linear correlations of IHC based expression (H-score).

	Expression in	Stroma	Stroma	Stroma	Neoplastic	Stroma	Neoplastic	Stroma	Inflammatory cells
Expression in		**ACTA2**	**TAGLN**	**CALD1**	**TPM2**	**TPM2**	**HEYL**	**HEYL**	**HEYL**
Stroma	**ACTA2**		0.38*	0.54*	0.12	0.29	0.03	0.1	-0.22
Stroma	**TAGLN**			0.3	0.1	0.12	0.08	0.1	0.02
Stroma	**CALD1**				-0.06	0.27	-0.2	0.1	-0.01
Tumor	**TPM2**					0.43*	0.52*	0.44*	0.39*
Stroma	**TPM2**						0.48*	0.62*	0.38*
Tumor	**HEYL**							0.41*	0.55*
Stroma	**HEYL**								0.48*
Inflammatory cells	**HEYL**								

Spearman rho values are shown (n = 40).
* $p < 0.05$

Table S15. Correlation of qRT-PCR based expression levels.

	ACTA2	CALD1	HEYL	TAGLN	TPM2	CDH1	VIM	IHC STROMA PERCENTAGE
**ACTA2**		0.89	0.57	0.95	0.84	-0.43	0.76	0.38
**CALD1**			0.62	0.9	0.87	-0.48	0.81	0.47
**HEYL**				0.5	0.57	-0.3*	0.64	0.3*
**TAGLN**					0.82	-0.43	0.77	0.41
**TPM2**						-0.45	0.7	0.4
**CDH1**							-0.41	-0.16*
**VIM**								0.42

Spearman rho values are shown for correlations between gene expression (n = 42) and correlations between gene expression and stromal percentage (n = 40).
* all relationships except these are significant ($p < 0.05$)
