# Supplementary material for: A Novel Gene List Identifies Tumors with a Stromal-Mesenchymal Phenotype and Worse Prognosis in Gastric Cancer
Source: Cancers (Basel). 2023 Jun 2;15(11):3035. doi: 10.3390/cancers15113035 (PMC10252086; doi:10.3390/cancers15113035)
Supplement: Supplementary file 1 [file cancers-15-03035-s001.zip › Supp Figure S10.pdf]

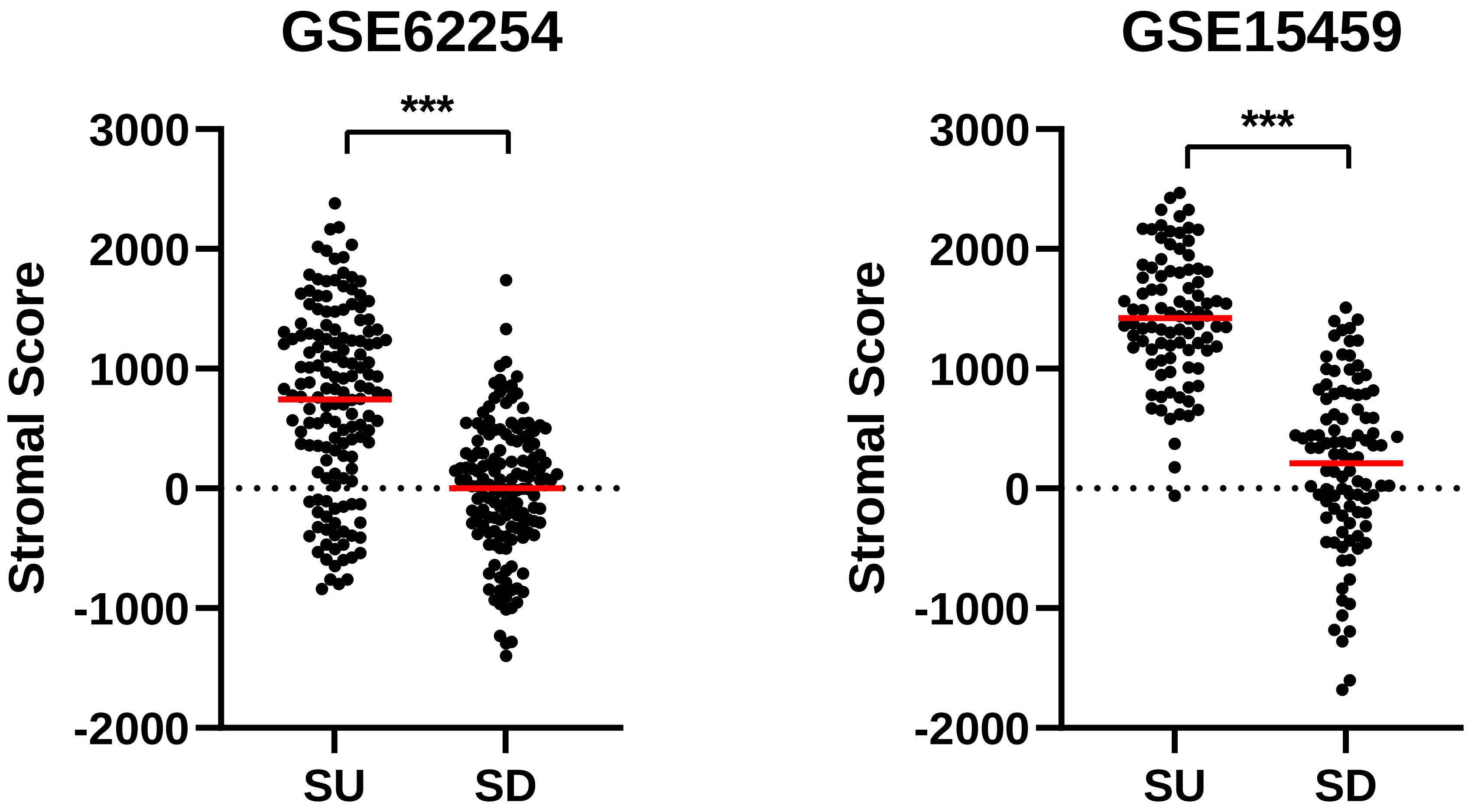

Supplementary Figure S10: Stromal scores based on ESTIMATE method for SU and SD samples in GSE62254 and GSE15459. Unpaired t-test p values are shown. *** P<0.0001
